# Fragment merging approach for design, synthesis, and biological assessment of urea/acetyl hydrazide clubbed thienopyrimidine derivatives as GSK-3β inhibitors

**DOI:** 10.1186/s13065-023-01026-w

**Published:** 2023-09-27

**Authors:** Joseph S. Saleh, Soha R. Abd El Hadi, Hany S. Ibrahim, Eman Z. Elrazaz, Khaled A. M. Abouzid

**Affiliations:** 1 Department of Pharmaceutical Chemistry, Faculty of Pharmacy, Egyptian Russian University, P.O. Box 11829, Badr City, Cairo, Egypt; 2 Department of Pharmaceutical Chemistry, Faculty of Pharmacy, Ain Shams University, P.O. Box 11566, Abbassia, Cairo, Egypt

**Keywords:** Thienopyrimidine, GSK3β, Gewald reaction, Acetyl hydrazide, Docking

## Abstract

New thienopyrimidine derivatives were designed and synthesized as GSK-3β inhibitors based on the structure of active binding site of GSK-3β enzyme. In this study, compounds **6b** and **6a** were found to be moderate GSK-3β inhibitors with IC_50s_ 10.2 and 17.3 μM, respectively. Molecular docking study was carried out by docking the targeted compounds in the binding site of the GSK-3β enzyme using the MOE program. Moreover, ADME study was performed to predict certain pharmacokinetic properties. The results showed that all synthesized compounds may not be able to penetrate the blood brain barrier; so, the chances of CNS side effects are predicted to be low. CYP1D6 is predicted to be inhibited by compounds (**5a**, **5d**, **6a**, **9a** and **9b**), So drug-drug interactions are expected upon administration of these compounds.

**Graphical Abstract:**

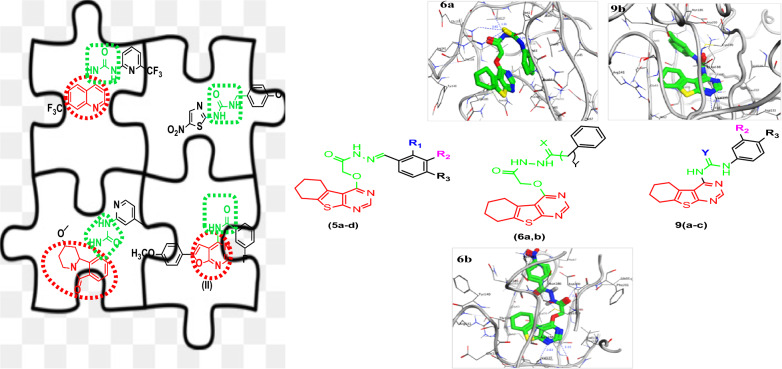

**Supplementary Information:**

The online version contains supplementary material available at 10.1186/s13065-023-01026-w.

## Introduction

Cancer is a genetic disease with two main characteristics: uncontrolled growth of the cells and tissue invasion/metastasis of the tissues [1] Cell cycle control is dependent on the four stages (G1, S, G2, M). A variety of metabolic pathways have been identified as critical processes for the start of a certain cell cycle event [2]. In these biochemical pathways, many protein kinases are active and employ regulated phosphorylation processes to relay biological information to downstream signaling pathways [3]. Glycogen Synthase Kinase-3 (GSK-3) is a multitasking serine/threonine protein kinase because of its broad participation in different signaling pathways [4]. GSK-3 is a cyclin-dependent kinase (CDK) proline-directed kinase, which also includes mitogen-activated protein kinases (MAPKs), cyclin-dependent kinases (CDKs), and CDK-like kinases (CLKs). GSK-3α and GSK-3β that encode 51 and 47 kDa proteins, respectively. Both isomers are found in cells and tissues, and their physiological activity is similar. The most common isoform is GSk3β which overexpressed in different human cancer, that why it considers as strong therapeutic target [5]. It is a kinase that belongs to the phosphotransferase family. Originally thought to control glycogen synthase, it is now shown to phosphorylate a huge variety of substrates, hence regulating a large range of biological activities, involving Wnt and Hedgehog signaling, transcription, and the insulin system [6]. Despite structural similarities between two isoforms, their roles are distinguished by phosphorylation at unique locations. For the activation of GSK 3α and GSK 3β, Tyr 279 and Tyr 216 are two sites situated at the T-loop of GSK 3 (activation domain) and are phosphorylated by upstream signaling molecules. Obviously, GSK 3α and GSK 3β are inhibited by site—specific phosphorylation, which is closely regulated by a variety of processes. All of these pathways for GSK3α inactivation at Ser21 and GSK 3β at Ser9 have been linked to the phosphoinositide3 kinase (PI3K) dependent mechanism. Activation of PI3 kinase, protein kinase A (PKA), and protein kinase B (PKB) (also termed as Akt), protein kinase C (PKC) and p90Rsk contribute to GSK3 inactivation. Inactivation of GSK 3β is also implicated in glycogen production, protein synthesis, cell invasion and cell proliferation. Consequently, GSK 3β is regarded as a possible therapeutic target in cancer therapy.

**Fig. 1:**
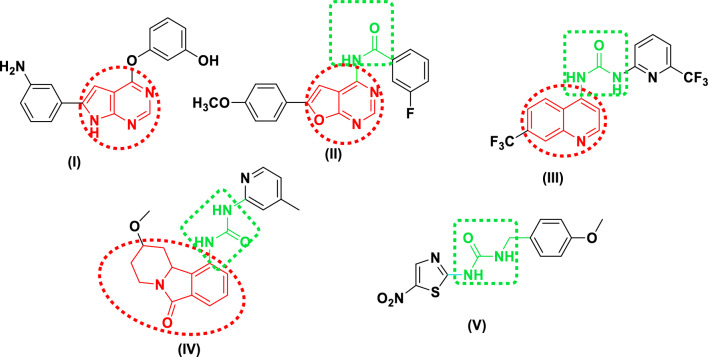
Some reported GSK3β inhibitors

Recently, many GSK-3β kinase inhibitors have been discovered that involve the quinazoline, quinoline, pyrrolopyrimidine, and furopyrimidine frameworks. These cores are thought to be a configuration that is preferred for suppressing ATP-dependent kinases. Pyrrolopyrimidine derivative (**I**) a promising GSK-3β inhibitor which inhibit proliferation and cytokine production in human lung cancer (IC_50_ = 30 nm) [7]. Moreover, another bioisosteric furopyrimidine derivative (**II**) shows IC_50_ > 35 µM on GSK-3β [8]. On the other hand, quinoline derivative (**III**) also inhibits GSK-3β with IC_50_ < 10 nm [9]. Tricyclic tetrahydropyridoisoindolone derivative (**IV**) as GSK-3β inhibitor with IC_50_ = 0.032 µM [10]. Also, thiazole derivative (**V**) containing urea moiety has shown IC_50_ = 2.7 nm against GSK-3β and used as antidepressants and also as mood stabilizer [11]. Motivated by the aforementioned facts, design novel GSK-3β inhibitors with higher activity is a promising targeted approach in treatment of cancer [12] (Fig. 1).

## Rationale and design

The structural study of GSK-3β inhibitors were observed certain interactions between to the ATP binding site via hydrogen bonds with Asp133 or Val135 (two key binding residues) [13]. Based on the urea moiety's substituent, we revealed two distinct mechanisms of binding. The first one, in the tetrahydropyridoisoindolone scaffold where the carbonyl group in one binding mode directs towards the catalytic Lys85, whereas the urea is parallel to the hinge area, forming a hydrogen bond contact between the urea and the backbone of Val135. In GSK3β, a second method of binding was more discovered. In the tetrahydropyridoisoindolone scaffold, the carboxyl group binds with the NH backbone of Val135 by a hydrogen link, and the catalytic Lys85 interacts with the urea via a hydrogen bond. If the ortho position contains nitrogen, the heterocyclic scaffold connected to the urea can form a further hydrogen bond with Lys85. The substituent connected to the saturated ring of the tetrahydropyridoisoindolone scaffold interacts with Thr138 through a hydrogen bond network or a van der Waals interaction, depending on the orientation of the residue side chain [14, 15]. According to the level of potency, activity of pyrrolopyrimidine (**I**), furopyrimidine (**II**), quinoline derivative (**III**) and tetrahydropyrido[1,2-a]isoindolone (**IV**) as GSK-3β inhibitors have IC_50_ in micromolar or nanomolar range, ring replacement of pyrrolopyrimidine and furopyrimidine with thienopyrimidine core to explore additional interaction with the hinge region of kinase.

**Fig. 2:**
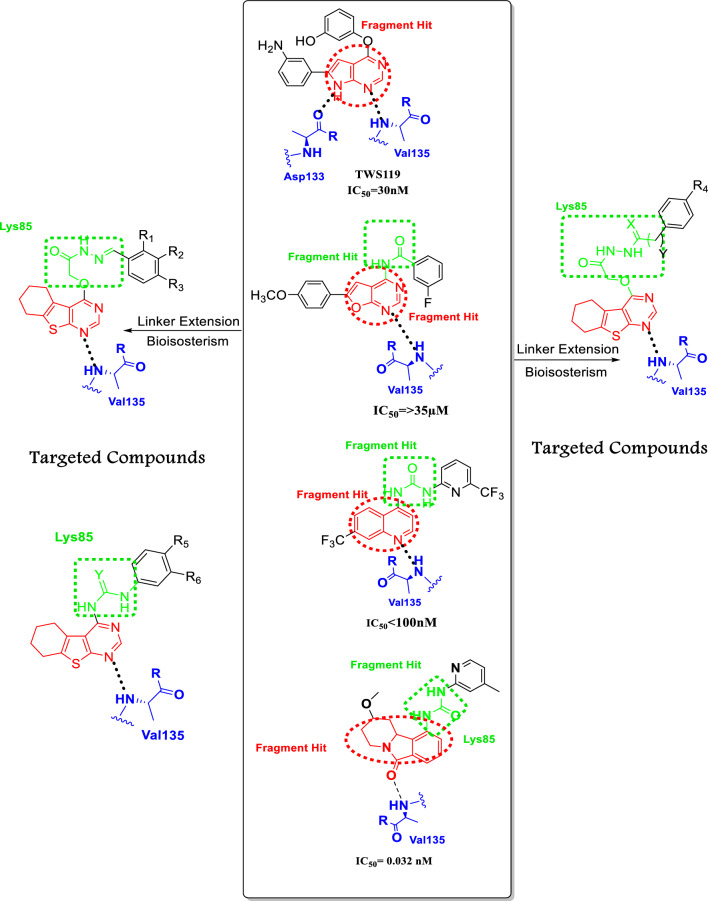
Schematic representation of targeted compounds

The goal of this work was to provide evidence to support the use of thienopyrimidine scaffold as a pharmacophoric constituent essential in the development of novel GSK-3β inhibitors [16]. In this study thienopyrimidine scaffold was utilized to design novel GSK-3β kinase inhibitor depending on the main reasons: novelty of this fragment because it is not exploited yet in any clinically approved drug. Also, tetrahydrothienopyrimidine can be readily synthesized from commercially available materials. Based on the aforementioned findings, fragment merging approach and structure extension through linking the urea moiety or amide moiety with thienopyrimidine scaffold to explore if occupy a deep hydrophobic allosteric pocket. As a result, several substitution patterns on (R) on the terminal phenyl ring were studied to investigate the interaction with the terminal allosteric pocket (Fig. 2).

## Results and discussion

### Chemistry

**Scheme 1:**
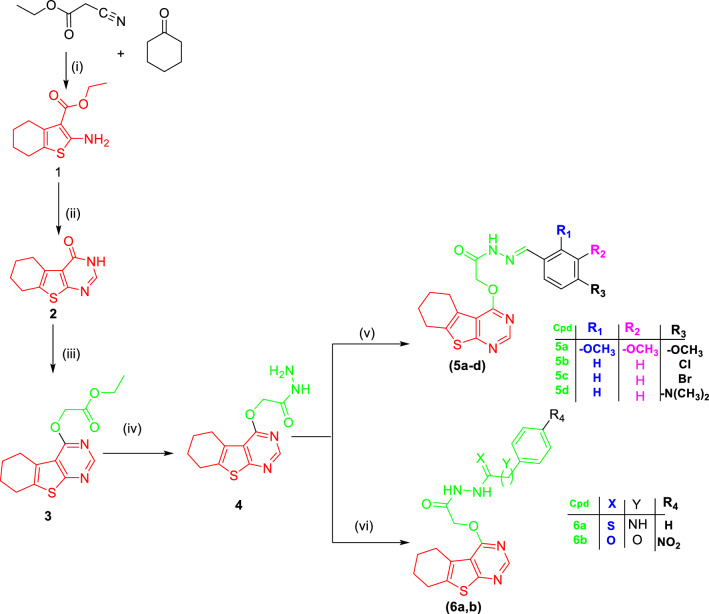
Reagent and conditions: (**i**) S^18^, piperidine, EtOH, RT, overnight (**ii**) HCONH_2_, reflux, 6 h. (**iii**) ClCH_2_COOC_2_H_5_, CS_2_CO_3_, DMF, 80 ℃, 24 h (**iv**) NH_2_NH_2_.H_2_O, CH_3_OH, 0 ͦ C, 4.5 h; (**v**) Ph-CHO derivatives, gl. AcOH, EtOH, reflux, 24–72 h (**vi**) *p*-NO_2_-Ph-COCl, TEA, dry toluene, reflux, 3 h or PhSCN, TEA, dry toluene, reflux, 48 h

**Scheme 2:**
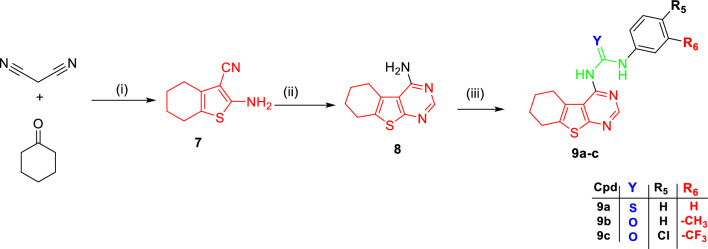
Reagent and conditions: (**i**) S^18^, TEA, EtOH, RT, overnight (**ii**) HCONH_2_, reflux, 5 h (**iii**) PhSCN/ PhCNO derivatives, CH_3_CN, reflux, 24–48 h

Scheme 1 offered synthesizing methods for targeted compounds. The appropriate intermediates, which were synthesized in following the below-mentioned techniques, were used to obtain final compounds containing a substituted urea moiety. Gewald reaction was used to synthesize ethyl 2-amino-4,5,6,7-tetrahydrobenzo[b]thienopyrimidine (**1**) which undergoes the typical Niementowski reaction to get 5,6,7,8-tetrahydrobenzo[4,5]thieno[2,3-d] pyrimidin-4(3H)-one (**2**) which reacts with ClCH_2_COOC_2_H_5_ in DMF at 80 °C for 24 h in the presence of Cs_2_CO_3_ to yield ester intermediates (**3**) [17–19]. Thienopyrimidine acetohydrazide (**4**) is synthesized by hydrazinolysis of ester derivative (**3**) [20]. Final compounds (**5a-d**) were synthesized by reacting thienopyrimidine acetohydrazide intermediate (**4**) with different substituted aldehydes in absolute EtOH and the catalytic quantity of gl. AcOH under reflux for 24–72 h to give the targeted compounds (**5a-d**) in poor yield % [21]. On the other hand, compounds (**6a-b**) were obtained by the reaction of thienopyrimidine acetohydrazide intermediate (**4**) with *p*-nitrobenzoyl chloride for 3 h [22]or phenyl isothiocyanate in dry toluene and catalytic amount of TEA under reflux for 48 h [23]. Preparation of the urea derivatives (**9a-c**) was achieved through Gewald reaction using malononitrile to afford intermediate (**7**) which was reacted with formamide to produce 4-aminothienopyrimidine intermediate (**8**). The previous intermediate reacts with different phenyl isocyanate or phenyl isothiocyanate to furnish urea derivatives (**9a-c**) (Scheme 2).

Several analytical and spectral approaches were used to understand the structural properties of the synthesized molecules. The ^**1**^**HNMR** spectra for the targeted compounds (**5a-d**, **6a**, **b** and **9a-c**) were consistent with total number of protons. ^**1**^**HNMR** spectra of compounds (**5a-d**) demonstrated two characteristic signals of the D_2_O exchangeable protons of NH groups around δ 11.48–11.94 ppm confirming the E/Z configuration. In the ^**1**^**HNMR** spectra of E/Z mixture, a peak at δ 7.92–8.39 ppm represented for N=**CH** as in compounds (**5a-d**) confirming the E/Z configuration with (3:1) ratio, where vinylic protons of (**5a-d**) appear at 8.39, 8.22, 8.21 and 8.07 ppm for the Z-isomer, whereas they appear at 8.24, 8.05, 8.04 and 7.92 ppm for the E-isomer. Intramolecular H-bonding between vinylic proton and the CO–NH proton leads to a downfield shift in all the vinylic protons in the Z-isomer and an upfield shift in the vinylic protons in the E-isomer. Additionally, the spectra showed one signal of O-**CH**_**2**_ showed as singlet around δ 5.19–4.70 ppm as in compound (**5a-d**) confirming the E/Z configuration. Additional protons were shown as a three singlet peaks around δ 3.85, 3.84 and 3.78 ppm of methoxy groups in compound (**5a**), also, singlet peak around δ 2.97 ppm for two methyl groups of -N(CH_3_)_2_ as in compound (**5d**). Furthermore, the extra signal of aromatic protons are around δ 6.75–7.77 ppm.

Regarding compound (**6a**) showed three signals around δ 10.62–9.34 ppm representing the D_2_O exchangeable proton of 3NH group while two signals around 10.93–10.64 ppm representing the D_2_O exchangeable proton of 2NH group in compound (**6b**). Additional aliphatic protons were shown in compounds (**6a**, **b**) as singlet peak around δ 4.73 and 4.82 ppm of the **O-CH**_**2**_ group. Also, the extra signal of aromatic protons are around δ 7.18–8.10 ppm.

On the other hand, ^**1**^**HNMR** spectra of compounds (**9a-c**) revealed the appearance of the 2 characteristic signals of D_2_O exchangeable protons of NH groups around δ 11.23 and 8.72 ppm. Regarding compounds (**9b**) spectra, showed singlet peaks at δ 3.06–2.27 ppm, respectively representing for the **CH**_**3**_ groups. In addition to the signal of aromatic protons are around δ 6.15–7.40 ppm.

In all synthesized compounds, protons were shown as a singlet peak at around δ 1.75–1.79 ppm of four protons at C_6_, C_7_ of cyclohexyl moiety, a singlet peak at around δ 2.74–2.78 ppm for two protons at C_5_ of cyclohexyl moiety, a singlet peak at δ around 2.80–2.88 ppm for two protons at C_8_ of cyclohexyl moiety. All spectra showed singlet peak of pyrimidine ring appear at around δ 8.34–8.29 ppm,

The ^**13**^**CNMR** spectra were consistent with number of carbons of the targeted compounds (**6a**, **6b**, **9a**, **9b**), where shown as a singlet peak at around δ 22.18, 22.84, 25.00, 25.76 (cyclohexyl), 167.18 ppm (C=O) for (**6a**), two C=O signals at 164.26 and 166.64 ppm for compound (**6b**). Both compounds (**6a**, **b**) showed a signal around δ 48.03–46.93 (O**CH**_**2**_). **IR** data stretching signal revealed of N–H group around 3371–3433 cm^−1^, aromatic C-H around 3000–3070 cm^−1^, aliphatic C–H at 2908–2989 cm^−1^, C=O or C=S around 1647–1708 cm^−1^ and C=N around 1600 cm-^1^. **Mass** analysis was performed on compounds showed the presence of M^+^, M^+^ + 1 and M^+^ + 2 peaks with A 3:1 relative intensity ratio refers to chlorine isotopes as compounds (**5b** and **9c**) while compound (**5c**) has relative intensity 1:1 corresponding to bromine isotopes. The founded molecular weights of the titled compounds were compared to their calculated molecular weights (Additional file 1: Sect. "Introduction".)

### Biological evaluation

#### GSK-3β serine/threonine kinase inhibitory activity *in vitro*

##### Preliminary screening at 100 µM )single dose Concentration)

The GSK-3β tyrosine kinase tests were carried out at Thermo Fischer Scientific USA (www.thermofischer.com/selectscreen). The investigation was carried out to evaluate the GSK-3β inhibitory activity of the synthesized compounds. During the main reaction of the kinase, a single residue of serine, threonine, or tyrosine in a produced FRET-peptide receives gamma-phosphate of ATP through the biological test Z'-LYTE. In the secondary process, Non-phosphorylated FRET-peptides are identified and degraded by a site-specific protease. The phosphorylation of FRET-peptides inhibits the development reagent from cleaving them. FRET between the donor (coumarin) and acceptor (fluorescein) fluorophores on the FRET-peptide is disrupted by cleavage, while FRET is maintained by uncleaved, phosphorylated FRET-peptides. The percentage of enzymatic activity that the tested substances inhibited GSK-3β kinase was compared to a 100 µM as reference concentration of the kinase inhibitor (Staurosporine with IC_50_ = 10 nM). As shown in Table 1, the compounds demonstrated moderate to outstanding inhibitory efficacy against GSK-3β kinases (Supplementary Material).

**Table 1:**
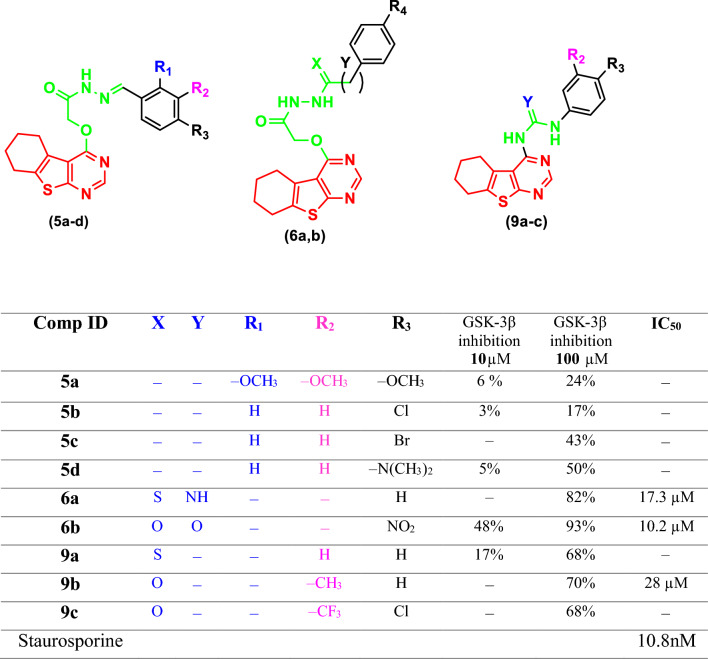
Percent inhibition against GSK-3β enzyme formed by the urea and amide-depend on analogues (**5a-d**, **6a-b** and **9a-c**)

##### Evaluation of five dose inhibitory activity (IC_50_)

Candidates which indicated appealing GSK3 inhibition % at 100 µM concentration (**6a**, **6b** and **9b**) were explored further for their dose-relationship at 5 various doses of enzymatic inhibition (10 nM–100 nM–1000 nM–10 µM–100 µM) after that calculate their IC_50_ values (Table 1).

**Fig. 3:**
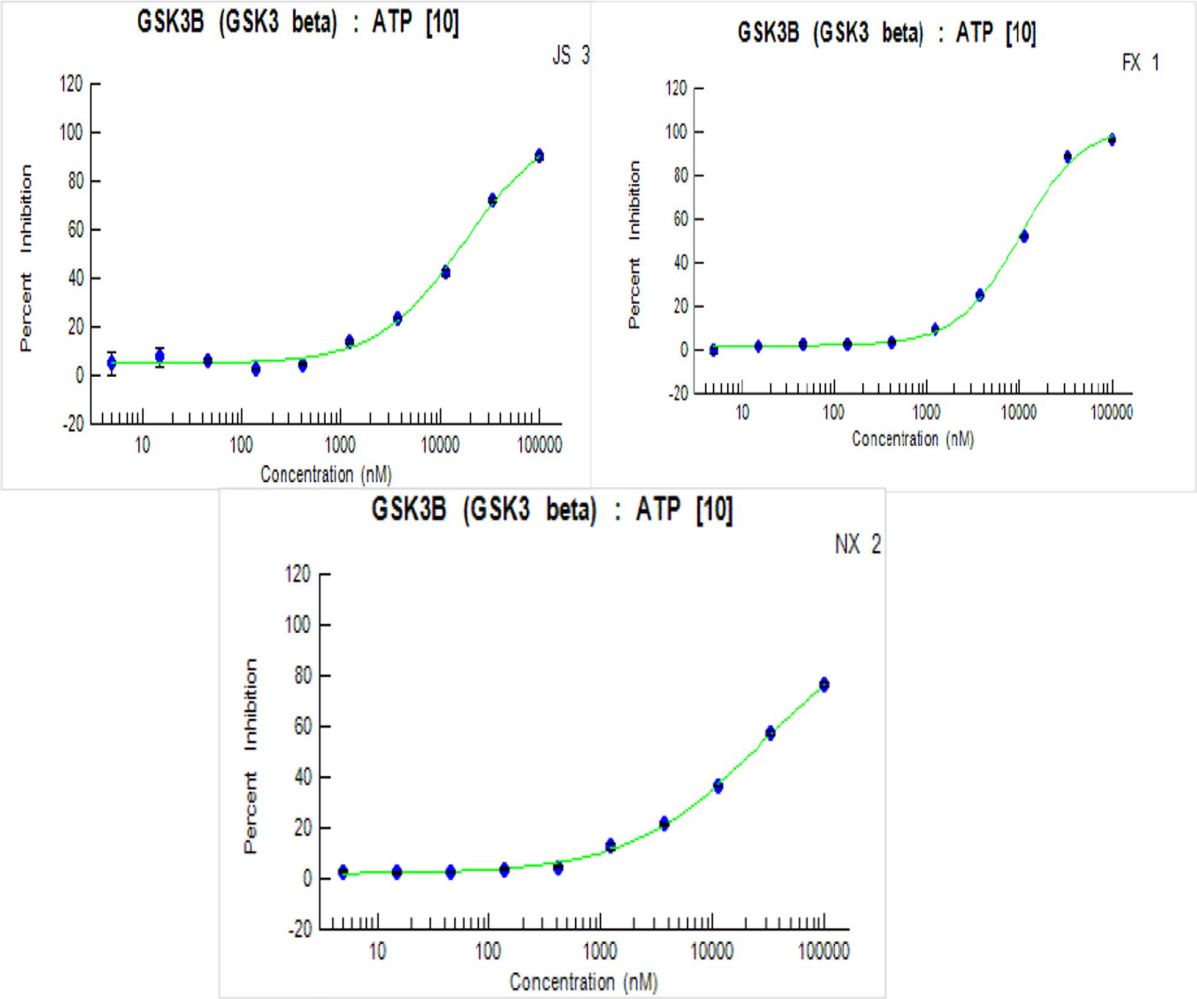
The target compounds' inhibitory concentration (IC_50_) on GSK-3 kinase activity

SAR study among the assessed thienopyrimidine derivatives with respect to the linker extension; it was found that the order of inhibitory activity seemed to be acetyl hydrazine-1-carbothioamide series (**6a-b**) more active than acetyl hydrazide series (**5a-d**). Unfortunately, other compounds showed poor inhibition on GSk-3β kinase. Nevertheless, the investigated thienopyrimidine derivatives (**9b-c**) with urea moiety either electron-donating or electron-withdrawing groups on terminal rings can be tolerated (Fig. 3).

##### Antiproliferative efficacy in vitro against NCI 60-cell line

The National Cancer Institute "NCI", NIH, Bethesda, Maryland, USA (www.dtp.nci.nih.gov) chose six of the final compounds for the Developmental Therapeutic Program (DTP), codes (**5a**, **5b**, **5c**, **5d**, **6a**, **6b**). The NCI screening service will favor compounds with drug-like modes of action based on computer-aided design. Selection The criterion for screening is the possibility of the submitted compounds to provide diversity to the NCI small molecule chemical collection. This screen employs 60 different Leukemia, melanoma, and additional tumor cell lines of the human from brain, kidney, lung, ovary, colon, breast, and prostate cancers. All compounds were picked based on their NCI codes. NSC: D-820696/1, NSC: D-820697/1, NSC: D-820698/1, NSC: D-820699/1, NSC: D-820700/1, NSC: D-820701/1. The initial 10 µM one dose percent inhibition assay was performed on the entire NCI 60 cell panel to examine the different chemotypes of this work. The results are presented as a percentage of cell growth on each of the 60 NCI cell line panels for each of the investigated substances in Table 2.

**Table 2:** Cell growth inhibition % of NCI 60 cancer cell lines displayed by studied final compounds (**5a, 5b, 5c, 5d, 6a, 6b**)

Cell line	Percent inhibition of tested compounds					
	**5a**	**5b**	**5c**	**5d**	**6a**	**6b**
**Leukemia**						
**CCRF-CEM**	− 1.41	− 9.27	− 6.6	− 3.06	− 9.95	− 4.24
*HL-60(TB)*	− 15.59	− 20.1	− 6.21	− 6.52	− 26.88	− 12.13
*K-562*	7.93	0.32	13.48	− 1.1	7.06	6.45
*MOLT-4*	− 2.51	− 3.88	9.38	0.81	− 8.69	− 5.68
*RPMI-8226*	− 2.02	2.71	− 4.46	− 1.59	3.86	3.54
*SR*	2.15	1.63	8.29	− 2.14	− 3.94	− 8.18
**Non-Small Cell Lung Cancer**						
*A549/ ATCC*	− 7.39	4.36	1.87	1.15	− 2.88	0.86
*EKVX*	− 2.52	3.11	3.66	− 0.83	− 1.87	− 2.61
*HOP-62*	− 0.97	− 5.2	− 7.64	− 2.81	− 3.51	− 5.83
*HOP-92*	2.18	0.71	− 17.11	− 12.12	− 5.18	− 6.04
*NCI-H226*	0.3	2.81	− 2.67	8.81	− 5.15	1.61
*NCI-H23*	− 3.2	− 3.09	0.25	− 7.75	3.1	− 3.5
*NCI-H322M*	− 2.38	0.39	− 5.25	3.69	0.95	− 2.3
*NCI-H460*	− 4.72	− 6.83	− 7.79	1.04	− 9.19	− 11.91
*NCI-H522*	5.39	− 7.4	10.29	4.74	5.09	7.64
**Colon Cancer**						
*COLO 205*	− 13.34	− 15.65	− 16.87	− 19.33	− 14.84	− 17.8
*HCT-116*	− 2.14	− 1.09	3.24	− 3.26	1.5	− 2.14
*HCT-15*	− 1.3	− 3.67	− 2.27	− 3.18	0.17	3.44
*HT29*	− 2.84	− 10.73	− 3.17	− 5.43	− 7.67	− 2.8
*KM12*	− 4.43	− 3.25	− 2.83	− 4.72	− 5.57	− 2.31
*SW-620*	− 0.67	− 2.86	− 2.19	2.39	− 2.97	− 0.18
**CNS Cancer**						
*SF-268*	0.3	4.27	5.08	1.44	− 1.63	− 1.73
*SF-295*	0.79	− 0.79	− 0.63	− 2.1	2.64	− 2.08
*SF-539*	5.93	− 1.8	1.02	− 4.86	2.42	2.53
*SNB-19*	6.88	9.77	5.53	3.31	5.27	9.18
*SNB-75*	28.47	18.43	13.6	18.79	14.57	20.29
*U251*	0.3	15.94	6.99	− 1.33	3.33	5.59
**Melanoma**						
*LOX IMVI*	4.82	7.09	3.68	2.93	1.31	4.71
*MALME-3 M*	2.96	6.05	− 6.85	− 6.83	− 5.31	3.05
*M14*	1.68	2	− 0.52	− 5.42	0.96	0.05
*MDA-MB-435*	− 3.78	− 4.44	0.85	− 1.25	− 2.38	− 2.51
*SK-MEL-2*	− 5.72	− 9.81	− 2.58	− 3.47	− 17.39	− 5.07
*SK-MEL-28*	− 3.83	− 2.26	− 8.66	− 1.38	− 5.56	− 0.83
*SK-MEL-5*	1.37	− 4.02	− 0.82	0.27	− 1.1	0.34
*UACC-257*	− 16.53	− 5.53	− 3.03	− 4.34	− 5.37	− 4.29
*UACC-62*	8.06	4.84	6.58	5.34	7.2	0.88
**Ovarian Cancer**						
*IGROV1*	1.47	11.04	2.52	14.45	− 2.19	1.22
*OVCAR-3*	− 10.52	− 16.96	− 7.55	− 4.24	− 11.84	− 12.61
*OVCAR-4*	− 1.36	− 7.63	− 8.18	− 7.86	− 8.59	− 4.28
*OVCAR-5*	5	8.96	10.01	8.97	3.88	0.17
*OVCAR-8*	− 9.71	− 3.32	− 4.36	− 4.59	− 1.94	− 2.71
*NCI/ADR-RES*	− 9.83	− 4.28	− 6.04	− 7.97	− 1.67	− 3.67
*SK-OV-3*	− 16.24	− 5.64	− 13.54	− 20.61	− 6.24	− 12.83
**Renal Cancer**						
*786–0*	0.81	2.11	5.22	− 7.8	1.23	0.04
*A498*	11.84	11.06	21.32	**48.37**	**46.98**	− 0.23
*ACHN*	0.55	− 2.86	− 5.34	− 2.45	− 3.69	− 2.45
*CAKI-1*	7.04	4.53	16.68	5.26	1.14	5.18
*RXF 393*	− 9.95	4.13	− 7.61	7.97	− 8.94	− 11.79
*SN12C*	5.91	2.55	3.09	− 0.68	3.79	2.5
*TK-10*	− 20.81	− 20.06	− 11.04	− 19.44	− 15.71	− 12.29
*UO-31*	23.31	23.08	16.55	23.98	14.76	21.12
**Prostate Cancer**						
*PC-3*	2.52	3.8	− 12.53	3.93	3.65	− 1.65
*DU-145*	− 5.76	− 6.67	− 0.13	− 4.77	− 8.64	− 5.56
**Breast Cancer**						
*MCF7*	10.68	11.7	8.67	7.82	12.27	8.21
*MDA-MB-231/ATCC*	6.61	7.6	11.25	− 1.8	3.71	− 2.07
*HS 578 T*	7.21	7.62	7.31	5.71	− 6.34	− 2.09
*BT-549*	− 1.41	− 5.46	2.58	− 9.47	− 2.68	− 0.91
*T-47D*	4.24	6.96	− 0.52	0.95	6.64	1.13
*MDA-MB-468*	− 13.63	− 8.56	− 14.65	15.75	− 14.73	− 5.3

40–50% inhibition of growth,  50–60% inhibition of growth,  60–70% inhibition of growth,  70–80% inhibition of growth,  80–100% inhibition of growth

**Results of in vitro NCI 60 cell panel assay**

On the entire NCI 60 cell panel, an initial in vitro one dose anticancer investigation was performed. The results for each compound were presented as a mean graph of the percentage growth of the treated cells compared to the untreated control cells. The mean graph of **5d** and **6a** results of the NCI 60 cell line screening program are shown in (Additional file 1: Section 2).

In the thieno[2,3-*d*]pyrimidine derivatives linked to the amide moiety (**5d**), and acyl hydrazide moiety as (**6a**) showed good anti-proliferative activity against specific renal cancer cell line (A498) with 48.37% and 46.98%, respectively (Table 2).

Only acyl hydrazide derivatives (**6b**) and (**6a**) showed moderate inhibitory activity on GSK-3β kinase with IC_50_s of 10.2 and 17.3 uM, respectively, these compounds may be a gloss of hope to develop more selective GSK-3β inhibitors in the future perspective.

### Molecular docking

In order to better understand the binding modalities and directions of active compounds into binding site of ATP for GSK-3β kinase enzyme, docking procedure is carried out using MOE software version 2019, and one of the ten retrievable docking configurations was picked. GSK-3β crystal structure in interaction with lead compound (**IV**) was obtained (PDB code: ***1J1B***). The binding modes of GSK-3β inhibitors showed that they interact with the ATP binding site via hydrogen bonding with Asp133 or Val135 which represent two out of the three interactions in all inhibitors namely: two hydrogen bond donors interaction with the backbone carbonyls of Val135 and Asp133, and one hydrogen bond acceptor interacts with Val 135's -NH. The ligands also interact hydrophobically with Ile62, Phe67, Leu188, and Cys199 side chains, as well as polarly with Thr138, Glu185, and Asp200.

#### Validation of docking protocol

**Fig. 4:**
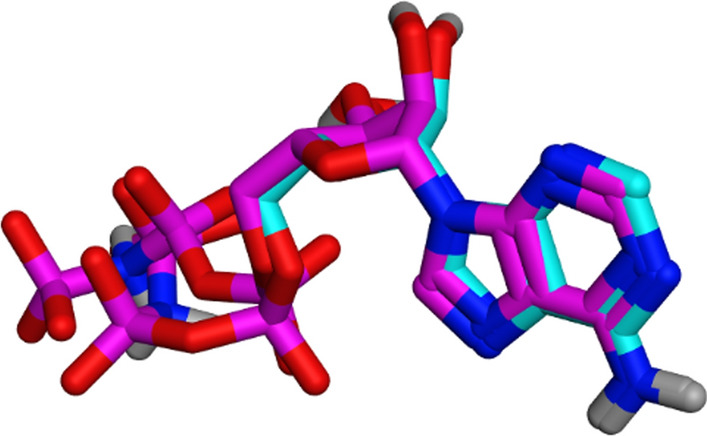
The aligning of the lead compound's X-ray active conformer (coloured in cyan) with the redocked posture (purple) at the GSK-3β binding site

Redocking phosphoaminophosphonic acid-adenylate ester (ANP) was used to validate the docking algorithm into the active site of GSK-3β. With a root mean square difference (RMSD) of 1.83 Å between the top docking pose and the original crystallographic ligand, this was found to be effective in retrieving the previously described X-ray crystal structure binding site of ligand (Fig. 4).

#### Binding mode of the targeted compounds with GSK-3β binding site (PDB ID: 1J1B)

See Fig. 5.

**Fig. 5:**
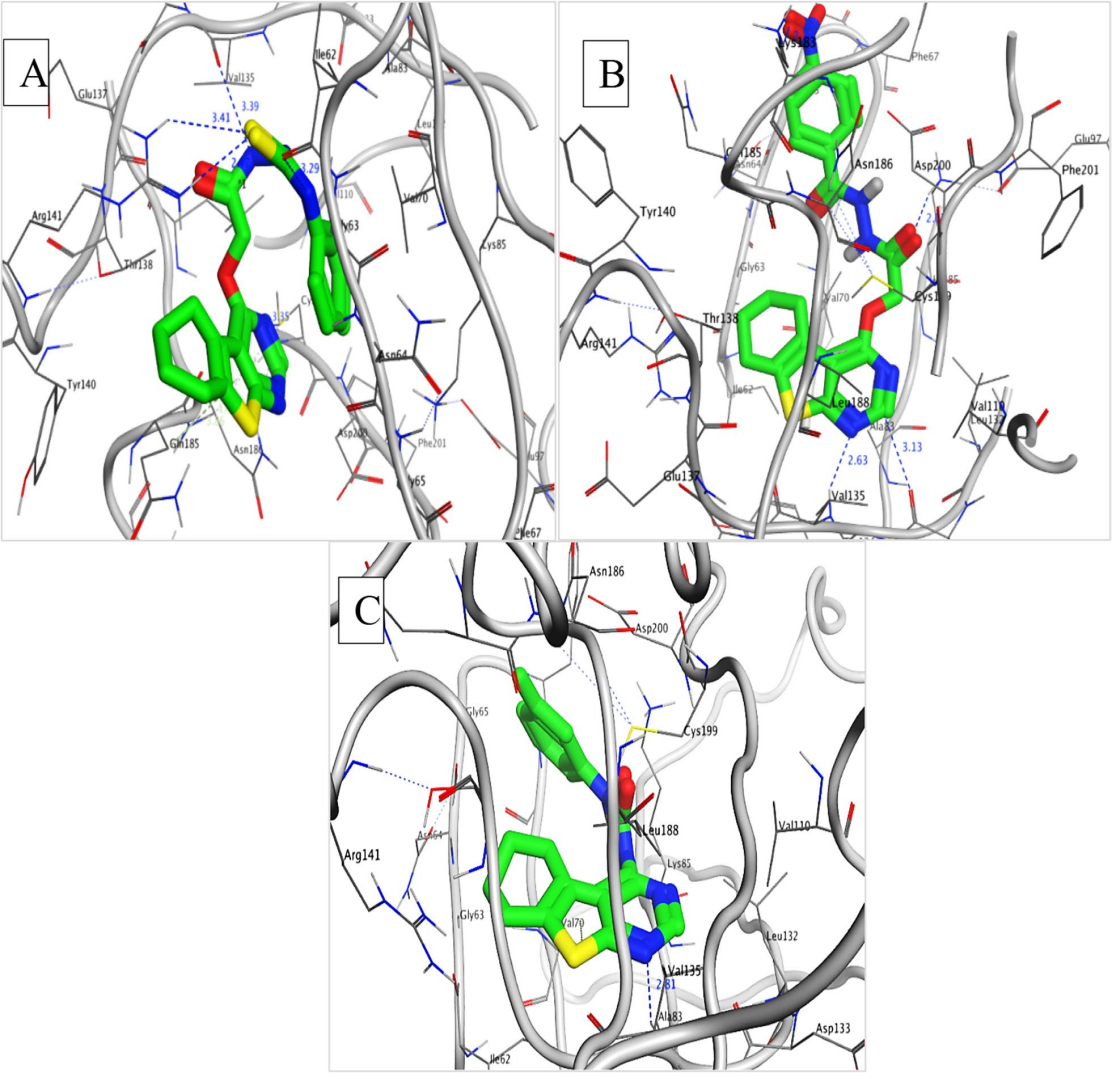
Targeted compounds bind with GSK-3β binding site (PDB ID: **1J1B**). Compound **6a** in ATP binding site of GSK-3β showing four hydrogen bond interactions with Val135, Arg141, Ille62, Cys 199 residues, and hydrophobic interaction with Gln185 while compound **6b** identified a critical hydrogen bond and its distances from the GSK-3β binding site; makes hydrogen bond interaction with Val135, Asp133, Lys85, Lys183 residues, and hydrophobic interaction with Val70 and Docking pose of compound **9b** show the same critical interactions with the GSK-3β binding site; hydrogen bond interaction with Val135 residue and hydrophobic interaction with Val70. (Dotted blue lines are represent for hydrogen bonds and pale green bold line for hydrophobic interaction)

### In silico predictive ADMET study

The pharmacodynamic properties of the synthesized compounds were studied in silico for the prediction of their possible transport to the site of action. Also, due to the importance of their molecular characteristics for human pharmacokinetics, molecular properties of new drugs were examined throughout development. For example, Lipinski's rule of five determines if a molecule of interest possesses chemical and physical qualities that would make it likely to be active orally. Oral bioavailability is likely to happen if at least three of the following requirements were met: There are a maximum of 5 H-bond donors, a maximum molecular weight of 500 Da, a maximum log P of 5, a maximum number of violations of 1 and a maximum number of H-bond acceptors of 10. The quantity of rotatable bonds, polar surface area, and drug oral bioavailability are all strongly correlated. According to the Veber rule, oral bioavailability for compounds with less than 10 rotatable bonds and a polar surface area (PSA) > 140 Å^2^ is acceptable. Additionally, ADME was determined using the SWISSADME program. Additionally, the SWISSADME made a prediction regarding the potential pharmacokinetic effects on a number of cytochromes P450 enzymes (CYP450), including CYP1A2, CYP2C19, CYP2C9, CYP2D6, and CYP3A4, as well as the likelihood that these enzymes will serve as substrates (inducers) of Permeability glycoprotein (P-gp) (Table 3).

**Table 3:** Predicted AMDE and pharmacological parameters for targeted compounds (**5a-d, 6a-b, 9a-c**)

Comp	M.wt (g/mol)	H. bond Donor	H. bond acceptor	Log p	TPSA	Lipinski violation	Rotatable bonds
**5a**	456.51	1	8	3.42	132.40 Å^2^	0	9
**5b**	400.88	1	5	4.01	104.71 Å^2^	0	6
**5c**	445.33	1	5	4.11	104.71 Å^2^	0	6
**5d**	409.50	1	5	3.56	107.95 Å^2^	0	7
**6a**	413.52	3	4	3.31	148.50 Å^2^	1	8
**6b**	427.43	2	7	2.21	167.27 Å^2^	1	8
**9a**	340.47	2	2	3.88	110.17 Å^2^	0	4
**9b**	338.43	2	3	3.67	95.15 Å^2^	0	4
**9c**	426.84	2	6	4.92	95.15 Å^2^		5

Comp	CYP1A2 inhibitor	CYP2C19 inhibitor	CYP2C9 inhibitor	CYP2D6 inhibitor	CYP3A4 inhibitor	P-gp substrate	BBB permeant
**5a**	NO	YES	YES	YES	YES	NO	NO
**5b**	YES	YES	YES	NO	YES	NO	NO
**5c**	YES	YES	YES	NO	YES	NO	NO
**5d**	NO	YES	YES	YES	YES	NO	NO
**6a**	YES	YES	YES	YES	YES	NO	NO
**6b**	NO	NO	NO	NO	YES	NO	NO
**9a**	YES	YES	YES	YES	YES	YES	NO
**9b**	YES	YES	YES	NO	YES	YES	NO
**9c**	YES	YES	YES	NO	YES	NO	NO

**Boiled-egg plot chart**

**Fig. 6:**
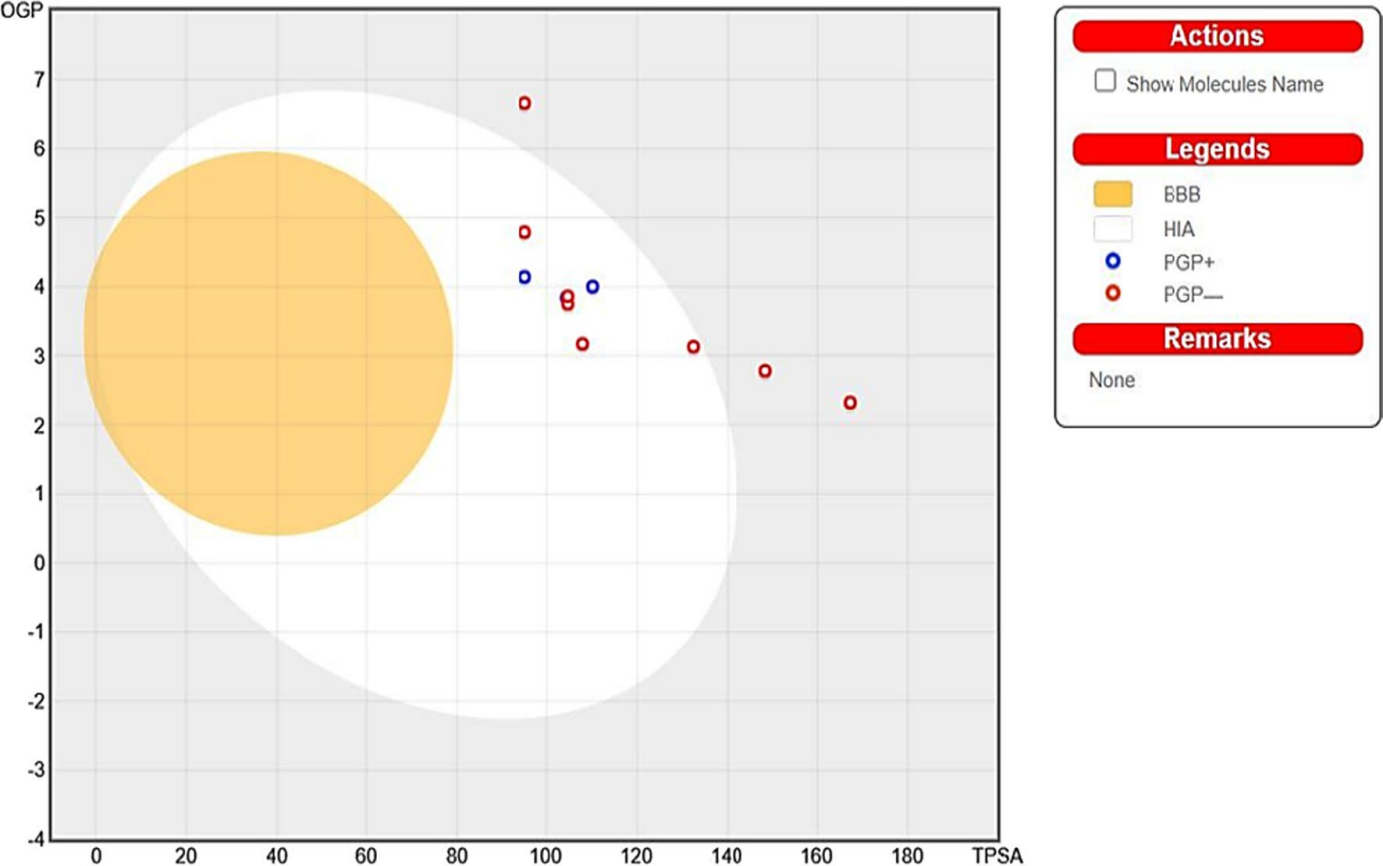
The newly synthesized compounds' Blood Brain Barrier (BBB) plot and human intestinal absorption (HIA)

ADMET-Graphic, a 2D plot created utilizing determined by Swiss ADME, is used to display the findings of the ADMET study. All the substances were plotted outside the in yellow circle that Blood Brain Barrier (BBB) and Human Intestinal Absorption (HIA) graphs some compounds are in white circle and 3 compound are outside (Fig. 6).

Pharmacokinetic analyses of compounds (**5a-d**, **6a**, **b** and **9a-c**) on cytochrome P450 enzymes and P-glycoprotein revealed that CYP3A4 is predicted to be inhibited by all compounds, and all compounds except compound (**6b**) are predicted to be inhibitors of CYP2C9 & CYP2C19, while CYP1D6 is predicted to be inhibited by compounds (**5a**, **5d**, **6a**, **9a** and **9b**) and all compounds, with the exception of those (**5a**, **5d** and **6b**)**,** are inhibitors of CYP1A2. Regarding P-gp, all substances are impermeable to glycoprotein except for (**9a-9b**). Additionally, the blood–brain barrier (BBB) is predicted to not be pass by all of the compound so it is expected to have no side effect on brain.

## Conclusion

This work involved the design and synthesis of new GSK-3β inhibitors containing urea /acetyl hydrazide coupled thienopyrimidine inhibitors. The synthesized compounds were examined for their in vitro GSK-3β inhibitory activities. Compounds **6a**, **6b**, **9a**, **9b** and **9c** were found to be moderately active as GSK-3β inhibitors with IC_50_ range (10.2–41.8 µM). In addition, six of the final compounds (**5a**, **5b**, **5c**, **5d**, **6a** and **6b**). The National Cancer Institute "NCI" chose them for a single dose screening programmed at 10 μM in the whole NCI 60 cell panel. The thienopyrimidine-based derivative (**5d** and **6a**) showed good anti-proliferative activity against specific cell lines as renal cancer cell line. Finally, molecular docking study was performed to predict how these compounds bind to the GSK-3β active site. Compounds (**6a**, **6b**, **9b**) showed similar orientation and binding interactions with a larger likelihood to enter the GSK-3β pocket. The study of computer aided ADMET was also carried out, using Swiss ADME to investigate the pharmacokinetic properties of the tested compounds.

## Experimental

### Chemistry

Loba chemie and alfa-aesar organics provided the starting ingredients and reagents, which were employed without further purification. Fisher Scientific or Sigma-Aldrich solvents were obtained and used without further purification. Drying of the solvent were done using molecular Sieves. TLC was used to monitor chemical reactions using silica gel 60 F254 packed on aluminium sheets purchased from Merck and viewed under ultraviolet light (λ = 254 nm). Silica (240–70 mm) was used for column chromatography. Stuart Scientific gear was used to determine melting points and were uncorrected. NMR spectra were acquired on a Bruker at 400 MHz for ^1^**HNMR** and at 100 MHz for ^**13**^**CNMR** in ppm scale. Chemical shifts (δH) were provided relative to DMSO-*d6*. All coupling constant (*J*) values were given in Hz. The acronyms are as follows: s: singlet; d: doublet; and m: multiplet. **IR** spectra were recorded on Schimadzu FT-IR 8400S spectrophotometer. An EI-MS LC/Ms/Ms mass spectrometer API 200 (AB Sciex Instrument) was utilized to get EI-MS spectra. Also, elemental analyses were also performed.

#### Ethyl 2-amino-4,5,6,7-tetrahydrobenzo[b]thiophene-3-carboxylate (**1**)

To a stirred solution of cyclohexanone (5.2 g, 53 mmol) in ethanol (15 mL), were added ethyl cyanoacetate (6.37 g, 57 mmol), sulfur powder (1.60 g, 50 mmol) and piperidine (1.3 g, 15 mmol) and the resulting reaction mixture was stirred at either 50 °C overnight in water bath. The progress of reaction was monitored by TLC, after completion of disappearance of starting materials, cooled the reaction mixture to ambient temperature and the resulting solids were filtered and dried under vacuum. The obtained crude solid was further purified by recrystallization by ethanol to afford desired compound-**1** (**9.76 g, yield: 80.1%**) as a yellow crystal [24].

#### 5,6,7,8-Tetrahydrobenzo [4,5] thieno[2,3-d] pyrimidin-4(3H)-one (**2**)

A solution of **1** (1 g, 4.4 mmol) in formamide (10 mL, 11.3 g, 250 mmol) was heated under reflux for 13 h. After cooling, the resulting solid precipitate was collected by filtration and recrystallized from ethanol to afford the titled compound-**2** (**0.72 g, yield: 78.6%**) as gray crystals [25].

#### Ethyl 2-((5,6,7,8-tetrahydrobenzo [4,5] thieno[2,3-d] pyrimidin-4-yl) oxy) acetate (**3**)

An equimolar mixture of 4,5,6,7-tetrahydrobenzo[b]thieno[2,3-d] pyrimidin-4(3H)-one (**2**) (6.18 g, 30 mmol), ethyl chloroacetate (3 mL, 30 mmol) and excess Cs_2_CO_3_ (6.5 g, 0.02 mol) in 30 mL of DMF was refluxed for 24 h. Most of the solvent was evaporated, and the reaction mixture was then poured onto ice water to give a solid product. Crystallization of the crude product from light petroleum (60–80 °C) yielded the title product **3** (**5.24 gm, yield: 64.1%**) as white crystals [26].

#### 2-((5,6,7,8-Tetrahydrobenzo [4,5] thieno[2,3-d] pyrimidin-4-yl) oxy) acetohydrazide (**4**)

A mixture of **3** (2.92 g, 10 mmol) and hydrazine hydrate (0.5 ml, 10 mmol) in methanol (60 mL) was heated under reflux for 4.5 h, left to cool. The formed precipitate was filtered, dried, and then recrystallized from ethanol to afford **4** (**1.61gm, yield: 61.1%**) as Pale-yellow crystals [26].

#### Synthesis of targeted compounds (**5a-d**)

**General procedure**

The hydrazide (**4**) (2.78 g,10 mmol) and the appropriate aromatic aldehyde, namely 2,3,4 trimethoxybenzaldehyde, *p*-chlorobenzaldehyde, *p*-bromobenzaldehyde, and dimethylaminobenzaldehyde (10 mmol), in ethanol (30 mL) and drops of acetic acid were heated under reflux for 48–72 h. A solid product was precipitated during reflux, which was filtered, washed well, dried and then recrystallized from a suitable solvent to afford **5a–d** (**yield: 61–64%**) (Spectral data in Supplementary Material).

##### 2-((5,6,7,8-Tetrahydrobenzo [4,5] thieno[2,3-d] pyrimidin-4-yl) oxy)-N'-(2,3,4-trimethoxybenzylidene) acetohydrazide (**5a**)

White powder, m.p 280–281 °C; yield: 61% (R_F_ = 0.64, Elution system: 1 ml of methylene chloride: 0.2 ml methanol: 0.4 ml hexane); ^**1**^**HNMR** (**400 MHz**, **DMSO-*d***_***6***_) δ 11.79, 11.64 (2s, 1H, NH D_2_O exchangeable, *Z* & *E* confirmations), 8.30 (s,1H, pyrimidine H), 8.39, 8.24 (2s, 1H, CH=N, *Z* & *E* confirmations (ratio 1:3)), 7.60, 7.55 (2d, *J* = 8.9 Hz, 8.8 Hz, 1H, ArH, *Z* & *E* confirmations), 6.92 (d, *J* = 8.0 Hz, 1H, ArH), 5.16, 4.71 (2s, 2H, CH_2_-O, *Z* & *E* confirmations), 3.84 (s, 3H, -OCH_3_), 3.85 (s, 3H, –OCH_3_), 3.78 (s, 3H, –OCH_3_), 2.86 (s, 2H, cyclohexyl), 2.78 (s, 2H, cyclohexyl), 1.79 (s, 4H, cyclohexyl); **FT-IR** (**ύ max, cm**^**−1**^): 3425 (NH), 3062 (aromatic CH), 2939 (aliphatic CH), 1681 (C=O); **MS** (Mwt.: 456): *m/z*, 456.60 [M^+^, (45.55%)], 280.48 (100%); **Anal**. Calcd for C_22_H_24_N_4_O_5_S: C, 57.88; H, 5.30; N, 12.27; Found: C, 58.12; H, 5.47; N, 12.50.

##### N'-(4-Chlorobenzylidene)-2-((5,6,7,8-tetrahydrobenzo [4,5] thieno[2,3-d] pyrimidin-4-yl) oxy) acetohydrazide (**5b**)

White powder, m.p 275–276 °C; yield: 64%, (R_F_ = 0.68, Elution system: 1 ml of methylene chloride: 0.2 ml methanol: 0.4 ml hexane);^**1**^**HNMR** (**400 MHz**, **DMSO-*d***_***6***_) δ 11,94, 11.84 (2s, 1H, NH, D_2_O exchangeable, *Z* & *E* confirmations), 8.30 (s, 1H, pyrimidine H), 8.22, 8.05 (2s, 1H, CH=N, *Z* & *E* confirmations (ratio 1:3)), 7.77 (d,* J* = 8.8 Hz, 2H, ArH), 7.52 (d, *J* = 8.8 Hz, 2H, ArH), 5.19, 4.74 (2s, 2H, CH_2_–O, *Z* & *E* confirmations), 2.86 (s, 2H, cyclohexyl), 2.76 (s, 2H, cyclohexyl), 1.78 (s, 4H, cyclohexyl); **FT-IR** (**ύ max, cm**^**−1**^): 3421 (NH), 3008 (aromatic CH), 2943 (aliphatic CH), 1685 (C=O); **MS** (Mwt.: 400): *m/z*, 400.41 [M^+^, (38.27%)], 401.85 [M^+^ + 2, (23.55%)], 47.77(100%); **Anal**. Calcd for C_19_H_17_ClN_4_O_2_S: C, 56.93; H, 4.27; N, 13.98; Found: C, 57.05; H, 4.41; N, 14.19. (Spectral data coincides with reported) [26].

##### N'-(4-Bromobenzylidene)-2-((5,6,7,8-tetrahydrobenzo[4,5]thieno[2,3-d]pyrimidin-4-yl)oxy)acetohydrazide (**5c**)

White powder, m.p 290–292 °C; yield: 62%; (R_F_ = 0.71, Elution system: 1 ml of methylene chloride: 0.2 ml methanol: 0.4 ml hexane); ^**1**^**HNMR** (**400 MHz, DMSO-*d***_***6***_) δ 11.85 (br. s, 1H, NH, D_2_O exchangeable), 8.30 (s, 1H, pyrimidine H), 8.21, 8.04 (2s, 1H, CH=N, *Z* & *E* confirmations (ratio 1:3)), 7.67 (m, 4H, ArH), 5.19, 4.74 (2s, 2H, CH_2_–O, *Z* & *E* confirmations), 2.86 (s, 2H, cyclohexyl), 2.76 (s, 2H, cyclohexyl), 1.78 (s, 4H, cyclohexyl); **FT-IR** (**ύ max, cm**^**−1**^): 3421 (NH), 3070 (aromatic CH), 2943 (aliphatic CH), 1666 (C=O); **MS**: (Mwt.: 445): *m/z*, 445.13 [M^+^, (36.68%)], 447.59 [M^+^ + 2, (35.03%)], 81.90 (100%); **Anal**. Calcd for C_19_H_17_BrN_4_O_2_S: C, 51.24; H, 3.85; N, 12.58; Found: C, 51.41; H, 3.98; N, 12.75.

##### N'-(4-(Dimethylamino) benzylidene)-2-((5,6,7,8-tetrahydrobenzo [4,5] thieno[2,3-d] pyrimidin-4-yl) oxy) acetohydrazide (**5d**)

Pale yellow powder, m.p 285 °C; yield: 64%; (R_F_ = 0.76, Elution system: 1 ml of methylene chloride: 0.2 ml methanol: 0.4 ml hexane); ^**1**^**HNMR** (**400 MHz, DMSO-*d***_***6***_) δ 11.56, 11.48 (2s, 1H, NH, D_2_O exchangeable, *Z* & *E* confirmations), 8.30 (s, 1H, pyrimidine H), 8.07, 7.92 (2s, 1H, CH=N, *Z* & *E* confirmations (ratio 1:3)), 7.53 (d, *J* = 8.9 Hz, 2H, ArH), 6.75 (d, *J* = 9.0 Hz, 2H, ArH), 5.14, 4.70 (2s, 2H, CH_2_–O,* Z* & *E* confirmations), 2.97 (s, 6H, 2-N–CH_3_), 2.86 (s, 2H, cyclohexyl), 2.76 (s, 2H, cyclohexyl), 1.79 (s, 4H, cyclohexyl); **FT-IR** (**ύ max, cm**^**−1**^): 3421 (NH), 3066 (aromatic CH), 2908 (aliphatic CH), 1678 (C=O); **MS**: (Mwt.: 409): *m/z*, 409.83 [M^+^, (90.98%)], 139.68 (100%); **Anal**. Calcd for C_21_H_23_N_5_O_2_S: C, 61.59; H, 5.66; N, 17.10; O, 7.81; S, 7.83; Found: C, 61.38; H, 5.79; N, 17.28.

#### Synthesis of targeted compounds (**6a, b**)

A mixture of (**4**) (0.17 g, 0.63 mmol) with phenyl isothiocyanate (0.17 g, 1.26 mmol) were dissolved in 15 ml dry toluene under reflux for 48 h and follow up the reaction with TLC till end the reaction, then cool to room temperature. A solid product was precipitated which was filtrated, dried to give compound **6a** (**yield 64%**) as white powder (Spectral data in supplementary material).

A mixture of compound (**4**) (0.3 g, 1.077 mmol) with 4-nitrobenzoyl chloride (0.2 g 1.077 mmol) was dissolved in 15 ml dry toluene and add drops of TEA in ice bath, stirring for 3 h and follow up the reaction with TLC till end the reaction. A solid product was precipitated during stirring, which was filtered off, washed well, dried and then recrystallized from ethanol to afford **6b** (**yield 64%**) as yellow powder (Spectral data in supplementary material).

##### N-Phenyl-2-(2-((5,6,7,8-tetrahydrobenzo [4,5] thieno[2,3-d] pyrimidin-4-yl)oxy)acetyl hydrazine-1-carbothioamide (**6a**)

White powder, m.p. 270–272 °C; yield: 64%; (R_F_ = 0.81, Elution system: 1 ml of methylene chloride: 0.2 ml methanol: 0.4 ml hexane); ^**1**^**HNMR** (400 MHz, DMSO-*d*_*6*_) δ 10.62 (s, 1H, NH, D_2_O exchangeable), 9.85 (s, 1H, NH, D_2_O exchangeable), 9.34 (s, 1H, NH, D_2_O exchangeable), 8.31 (s, 1H, pyrimidine H), 7.56 (d, *J* = 8.0 Hz, 2H, ArH), 7.34 (t, *J* = 8.0 Hz, 2H, ArH), 7.18 (t, *J* = 8.0 Hz, 1H, ArH), 4.73 (s, 2H, O-CH_2_), 2.80 (s, 2H, cyclohexyl), 2.76 (s, 2H, cyclohexyl), 1.80(s, 4H, cyclohexyl).^**13**^**CNMR** (**100 MHz, DMSO-*d***_**6**_) δ 22.18, 22.84, 25.00, 25.76 (cyclohexyl), 48.03 (CH_2_), 122.09, 125.63, 128.59, 131.14, 133.92, 139.34, 148.28, 157.88, 162.49, 167.18 (C=O),180.88 (C=S); **FT-IR** (**ύ max, cm**^**−1**^): 3414, 3329, 3221 (3NH), 3028 (aromatic CH), 2927(Aliphatic CH), 1670 (C=O); **MS**: (Mwt: 413): *m/z*, 413.57 [M^+^, (45.19%)], 109.31 (100%); **Anal**. Calcd for C_19_H_18_N_4_O_2_S_2_: C, 55.19; H, 4.63; N, 16.94; Found: C, 55.45; H, 4.72; N, 17.12.

##### 4-Nitro-N'-(2-((5,6,7,8-tetrahydrobenzo [4,5] thieno[2,3-d] pyrimidin-4-yl) oxy) acetyl benzo hydrazide (**6b**)

Yellow powder, m.p. 320–321 °C; yield: 64%; (R_F_ = 0.41, Elution system: 1 ml of methylene chloride: 0.2 ml methanol: 0.4 ml hexane); ^**1**^**HNMR** (**400 MHz, DMSO-*d***_***6***_) δ 10.93 (s, 1H, NH, D_2_O exchangeable), 10.64 (s, 1H NH, D_2_O exchangeable), 8.34 (m, 3H, 2ArH,1H pyrimidine H), 8.10 (d, *J* = 8.8 Hz, 2H, ArH), 4.82 (s, 2H O-CH_2_), 2.88 (s, 2H, cyclohexyl), 2.74 (s, 2H, cyclohexyl), 1.75 (s, 4H, cyclohexyl); ^**13**^**CNMR** (**100 MHz, DMSO-*d***_**6**_) δ 22.23, 22.87, 25.00, 25.77 (cyclohexyl), 45.89 (CH_2_), 122.10, 124.19, 129.50, 129.71, 131.18, 131.27, 133.55, 138.35, 148.55, 149.87, 157.27, 162.16, 164.26, 166.64 (C=O); **FT-IR** (**ύ max, cm**^**−1**^): 3429,3209 (2NH), 3105 (aromatic CH), 2974 (aliphatic CH), 1716, 1651 (C=O); **MS**: (Mwt.: 427): *m/z*, 427.47 [M^+^, (29.71%)], 142.21 (100%). **Anal**. Calcd for C_19_H_17_N_5_O_5_S: C, 53.39; H, 4.01; N, 16.38; Found: C, 53.51; H, 4.24; N, 16.59.

#### Amino-4,5,6,7-tetrahydrobenzo[b]thiophene-3-carbonitrile (**7**)

A mixture of cyclohexanone (1.85 g, 1.96 mL, 19.0 mmol), malononitrile (1.32 g, 1.25 mL, 57 mmol), sulfur powder (0.64 g, 20.0 mmol) and triethylamine (2.03 g, 2.12 mL, 20 mmol) was heated with ethanol (30 mL) in water bath at 50–60 °C overnight. After cooling, the resulting crystals was collected by filtration to give the titled compound (**7**) (**3.05 g, yield: 89.05%**) as buff crystals [27].

#### 5,6,7,8-Tetrahydrobenzo [4,5] thieno[2,3-d] pyrimidin-4-amine (**8**)

A mixture of 2-amino-4,5,6,7-tetrahydrobenzo[b]thiophene-3- carbonitrile (**7**) (1.78 g, 10.0 mmol) and formamide (3.60 g, 80.0 mmol) was refluxed for 5 h. On cooling, a precipitate crystallized, which was filtered off to produce (**8**) (**1.76 g Yield: 86%**) as orange crystals [28].

#### Phenyl-3-(5,6,7,8-tetrahydrobenzo [4,5] thieno[2,3-d] pyrimidin-4-yl) thiourea (**9a-c**)

*General procedure* A mixture of compound **8** (0.63 mmol, 0.129 gm) and appropriate phenyl isothiocyanate/ phenyl isocyanate derivative (1.26 mmol) in 10 mL acetonitrile was heated under reflux for 24–48 h. After cooling, the resulting crystals was collected by filtration, dried and recrystallized from ethanol to afford (**9a-c**) (**Yield 80–85%**) [29] (Spectral data in supplementary material).

##### Phenyl-3-(5,6,7,8-tetrahydrobenzo [4,5] thieno[2,3-d) pyrimidin-4-yl) thiourea (**9a**)

Yellow powder, m.p 300 °C; Yield: 84%, (R_F_ = 0.40, Elution system: 1 mL of methylene chloride: 0.15 mL methanol: 0.5 ml hexane); ^**1**^**HNMR** (**400 MHz, DMSO-*d***_***6***_) δ 10.41 (s, 1H, NH D_2_O exchangeable), 8.39 (s, 1H, NH D_2_O exchangeable), 8.17 (s, 1H, pyrimidine H), 7.36 (m, 3H, ArH), 7.13 (m, 1H, ArH), 6.77 (m, 1H, ArH), 2.91 (s, 2H, cyclohexyl), 2.75 (s, 2H, cyclohexyl), 1.81 (s, 4H, cyclohexyl); ^**13**^**CNMR** (**100 MHz, DMSO-*d***_**6**_) δ 22.69, 22.91, 25.30, 25.88 (cyclohexyl), 115.49, 124.04, 124.63, 127.37, 129.21, 129.88, 131.42, 132.14, 139.00, 151.46, 153.34, 158.56, 161.42, 165.89, 166.23 (C=S); **FT-IR** (**ύ max, cm**^**−1**^): 3371, 3317 (2NH), 3055 (aromatic CH), 2927 (aliphatic CH); **MS**: (Mwt.: 340): *m/z*, 340.75 [M^+^, (25.15%)], 85.85 (100%). **Anal**. Calcd for C_17_H_16_N_4_S_2_: C, 59.97; H, 4.74; N, 16.46; Found: C, 60.23; H, 4.86; N, 16.73.

##### 1-(5,6,7,8-Tetrahydrobenzo[4,5]thieno[2,3-d]pyrimidin-4-yl)-3-(m-tolyl)urea (**9b**)

Light brown powder, m.p. 300 °C Yield: 85%; (R_F_ = 0.44, Elution system: 1 mL of methylene chloride: 0.15 mL methanol: 0.5 mL hexane); ^**1**^**HNMR** (**400 MHz, DMSO-*d***_***6***_) δ 11.23 (s, 1H, NH D_2_O exchangeable), 8.71 (s, 1H, NH D_2_O exchangeable), 8.28 (s, 1H, pyrimidine H), 7.43 (d, *J* = 8.3 Hz, 1H, ArH), 7.24 (d, *J* = 7.2 Hz, 1H, ArH), 6.91 (d, *J* = 7.5 Hz, 2H, ArH), 3.05 (s, 3H, cyclohexyl), 2.84 (s, 2H, cyclohexyl), 2.31 (s, 3H, CH_3_), 1.83 (s, 4H, cyclohexyl); ^**13**^**CNMR** (**100 MHz, DMSO-*d***_**6**_) δ 21.67 (CH_3_), 22.44, 22.69, 25.29, 25.89 (cyclohexyl), 115.77, 117.06, 119.12, 120.33, 122.94, 127.33, 129.03, 131.43, 138.38, 140.12, 153.32, 158.56, 165.91(C=O); **FT-IR** (**ύ max, cm**^**−1**^): 3429, 3201 (2NH), 3028 (aromatic CH), 2978 (aliphatic CH), 1708 (C=O); **MS**: (Mwt.: 338): *m/z*, 338.30 [M^+^, (47.36%)], 262.84 (100%); **Anal**. Calcd for C_18_H_18_N_4_OS: C, 63.88; H, 5.36; N, 16.56; O, 4.73; Found: C, 64.12; H, 5.49; N, 15.75.

##### 1-(4-Chloro-3-(trifluoromethyl)phenyl)-3-(5,6,7,8tetrahydrobenzo[4,5]thieno[2,3-d]pyrimidin-4-yl)urea (**9c**)

Gray powder; m.p. 300 °C, Yield: 80%; (R_F_ = 0.33, Elution system: 1 mL of methylene chloride: 0.15 mL methanol: 0.5 mL hexane) ^**1**^**HNMR** (**400 MHz, DMSO-*d***_***6***_) 11.25 (s, 1H, NH, D_2_O exchangeable), 8.17 (s, 1H, pyrimidine H), 7.26 (d, *J* = 8.6 Hz, 1H, ArH), 6.99 (s, 1H, ArH), 6.78 (m, 2H, ArH, NH D_2_O exchangeable), 2.90 (s, 2H, cyclohexyl), 2.74 (s, 2H, cyclohexyl), 1.81 (s, 4H, cyclohexyl). **FT-IR** (**ύ max, cm**^**−1**^): 3433,3167 (2NH), 3039 (aromatic CH), 2947 (aliphatic CH), 1708 (C=O); **MS**: (Mwt.: 426): *m/z*, 426.46 [M^+^, (100%)], 428.35 [M^+^ + 2, (17.14%)]. **Anal**. Calcd for: **C**_**18**_**H**_**18**_**N**_**4**_**O**_**2**_**S**: C, 50.65; H, 3.31; N, 13.13; Found: C, 50.89; H, 3.45; N, 13.41.

### Biological assessment

#### In vitro GSK-3β inhibitory assays

The assay was run to gauge the designed compounds' ability to inhibit GSK-3. The biochemical test Z'-LYTE A synthesized FRET-peptide receives the gamma-phosphate of ATP produced by the kinase during the main reaction at a single tyrosine, serine, or threonine residue. Non-phosphorylated FRET-peptides are found and degraded by a site-specific protease in the secondary reaction. Development Reagent cleavage is prevented by FRET-peptide phosphorylation. While FRET is maintained by uncleaved, phosphorylated FRET-peptides, FRET between the donor (coumarin) and acceptor (fluorescein) fluorophores on the FRET-peptide is broken by cleavage. The radiometric approach, which quantifies reaction progress, determines the ratio (the Emission Ratio) of donor emission to acceptor emission following excitation of the donor fluorophore at 400 nm. By calculating how much ATP is still in solution after a kinase reaction, it quantifies kinase activity. The quantity of ATP present and the degree of kinase activity are both negatively connected with the luminous signal from the experiment.

#### Assay for anti-proliferative action against a range of NCI 60-cell lines in vitro

The NCI cancer screening procedure includes a comparison of all substances to the sixty NCI cell line panel representing leukemia, NSCLC, cell lines from breast cancer, prostate cancer, melanoma, CNS cancer, renal cancer, ovarian cancer, and colon cancer at a single point of 10 uM. The results of the single-dose screen can be presented using a mean graph.

### Molecular modeling

#### Molecular docking

**Protein preparation**

The protein data bank was utilized to get the structure of the GSK3-β proteins, which has (PDB ID: ***1JIB***). Partial charges were calculated and the enzymes were protonated. In the simulations, water molecules surrounding the cocrystallized ligands were deleted. The defined and isolated binding pocket was determined.

**Ligand preparation**

The following steps are taken by test ligand structures to prepare them for docking: The target molecules were created using ChemBioDraw Ultra 14.0, which was then repeated to MOE. (2) In three dimensions, the chemicals were protonated. (3) The constructions were built to use as little energy as possible The Merk Molecular Force Field produces a gradient of 0.5 (MMff94x). (4) Each molecule's force field partial charges were calculated. (5) Each molecule was subjected to a stochastic conformational analysis using default parameters, and the results were saved in a separate conformational database. (6) The most stable conformers of each molecule were recorded in a separate database in order for each molecule to dock onto the active site of the androgen receptor.

#### In silico ADMET study

Measuring drug probability and pharmacokinetics properties is critical in the development of novel medications. The Swiss Institute of Bioinformatics' SWISSADME server, a free web service, our target compounds' physicochemical characteristics were computed using ' ADME characteristics, pharmacokinetic qualities, and druglike nature may all be predicted.

### Supplementary Information

**Additional file 1**. supplementary material.

## Data Availability

All data generated or analyzed during this study are included in this published article (and its additional information files).
