# Supplementary material for: Fragment merging approach for design, synthesis, and biological assessment of urea/acetyl hydrazide clubbed thienopyrimidine derivatives as GSK-3β inhibitors
Source: BMC Chem. 2023 Sep 27;17(1):127. doi: 10.1186/s13065-023-01026-w (PMC10538245; doi:10.1186/s13065-023-01026-w)

**Fragment merging approach for design, synthesis, and biological assessment of urea/acetyl hydrazide clubbed thienopyrimidine derivatives as GSK-3β inhibitors**

**Supplementary materials.**

**Section 1. Some Spectroscopic charts [^1^HNMR, ^13^CNMR, IR, Mass] for some synthesized compounds**

#### 2-((5,6,7,8-Tetrahydrobenzo [4,5] thieno[2,3-d] pyrimidin-4-yl) oxy)-N'-(2,3,4-trimethoxybenzylidene) acetohydrazide (5a)

**^1^HNMR chart (5a)**


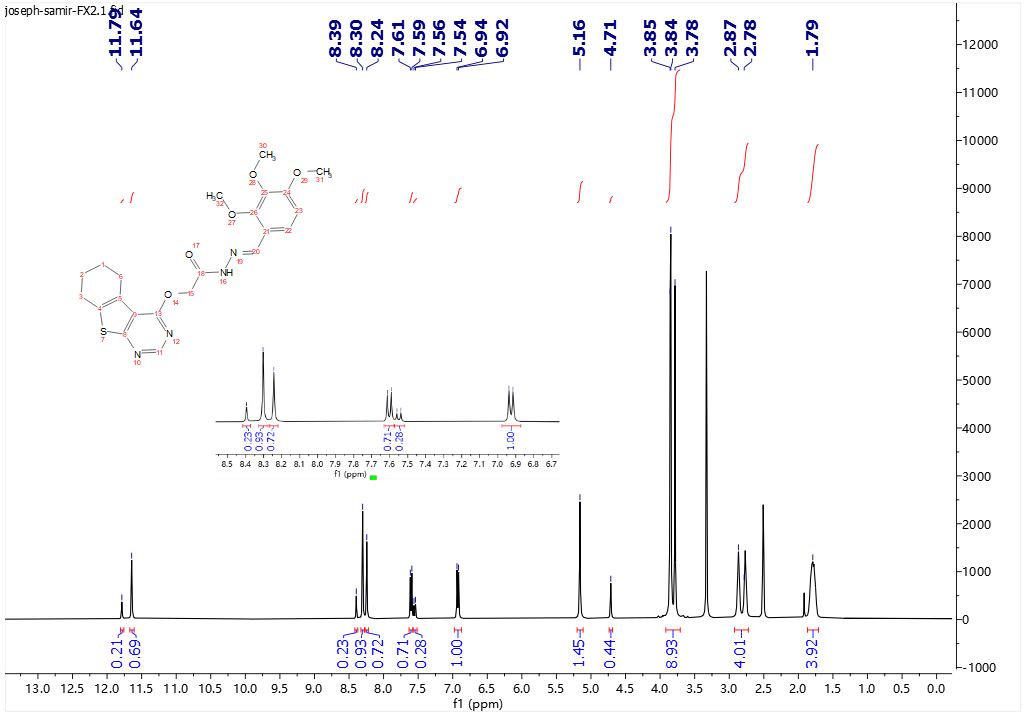


**D_2_O proton NMR (5a)**


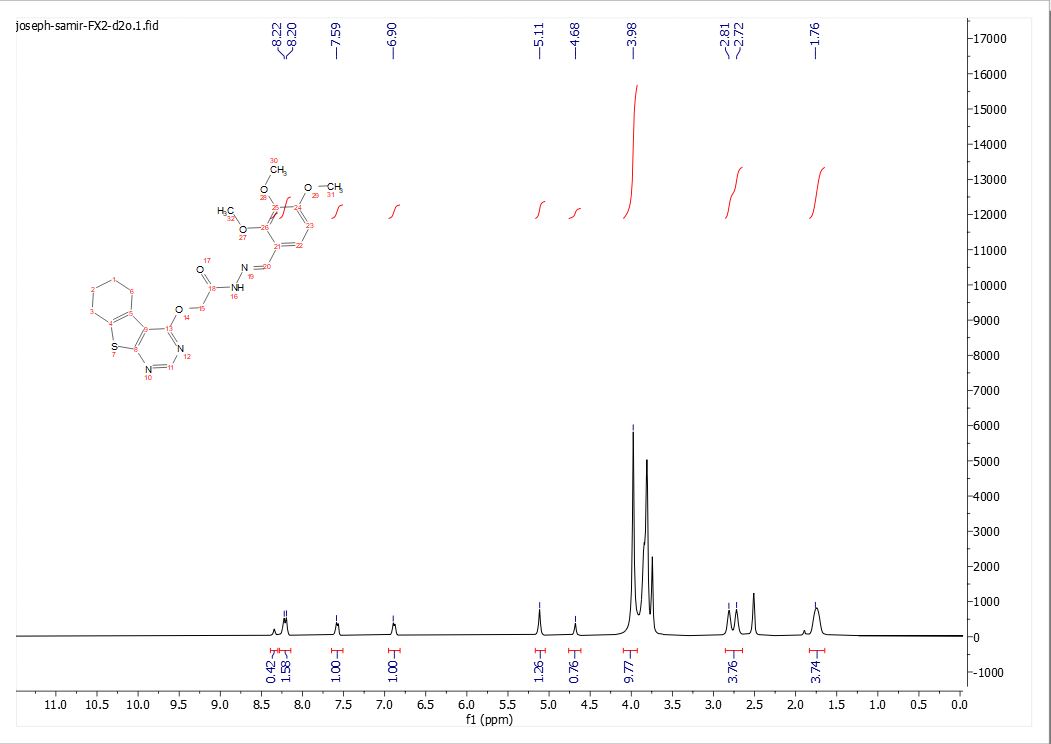


#### N'-(4-Chlorobenzylidene)-2-((5,6,7,8-tetrahydrobenzo [4,5] thieno[2,3-d] pyrimidin-4-yl) oxy) acetohydrazide (5b)

**^1^HNMR chart (5b)**


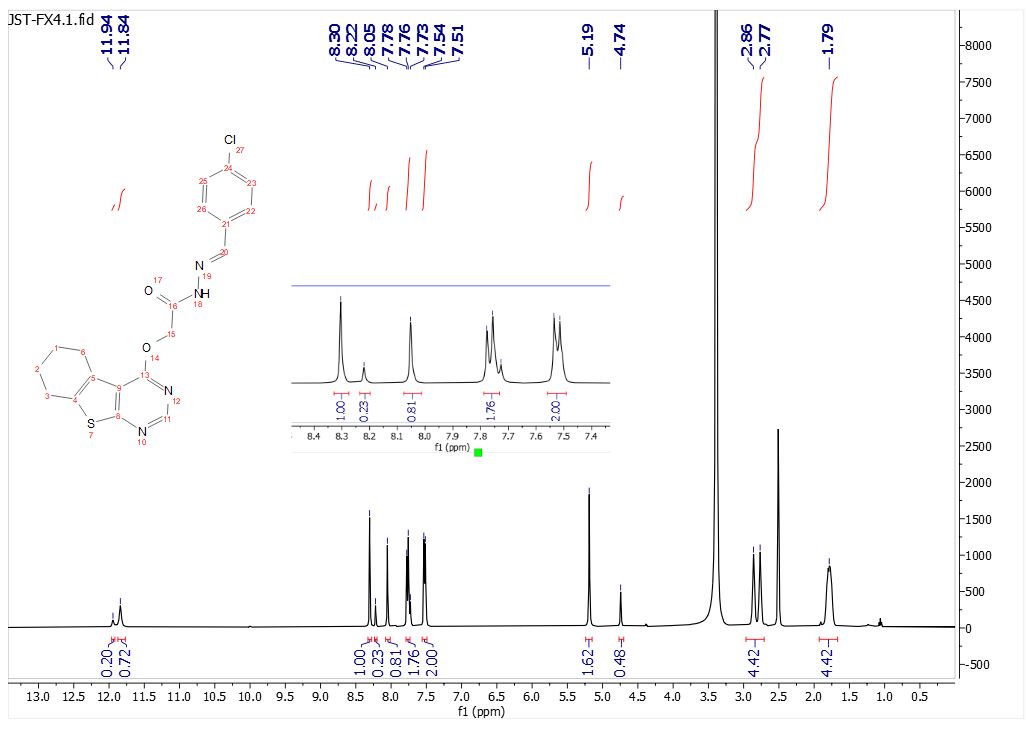


**IR chart (5b)**

**
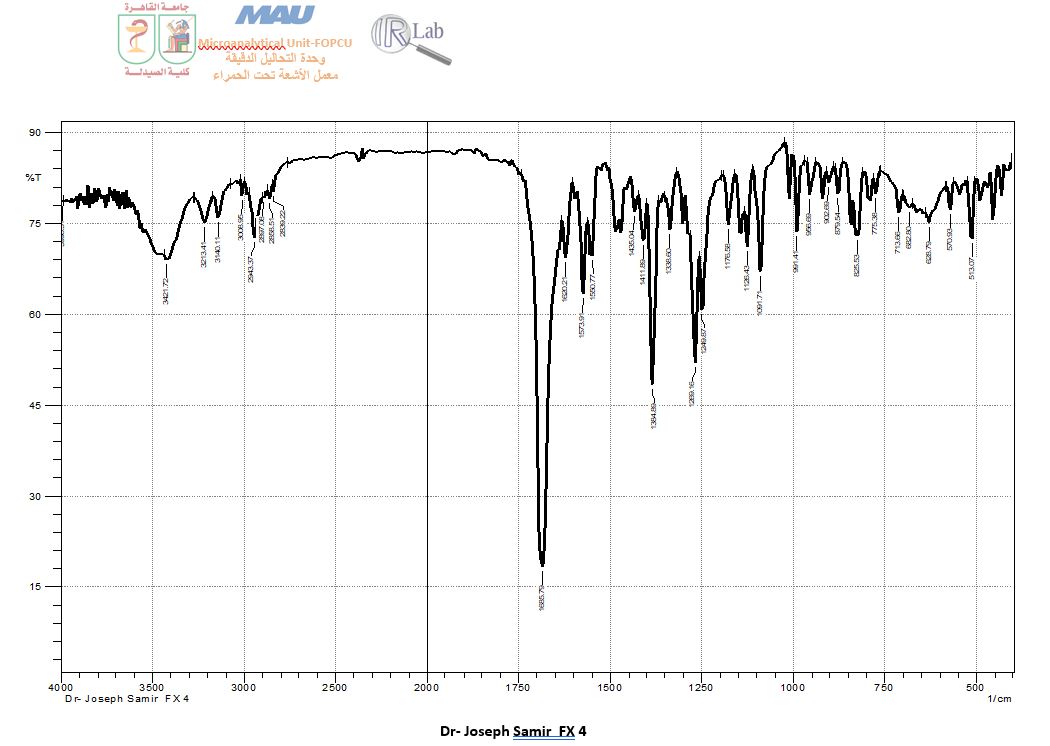
**

**Mass chart (5b)**


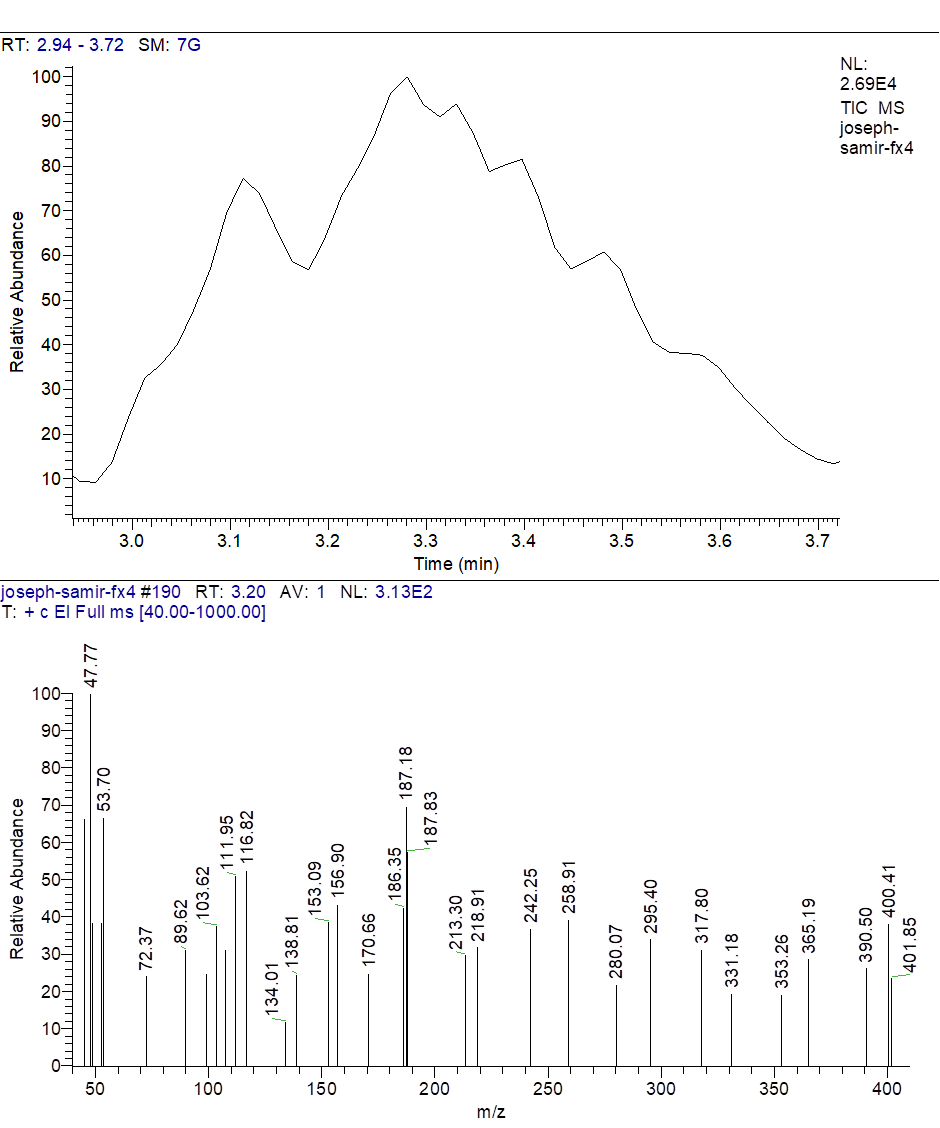


#### N'-(4-Bromobenzylidene)-2-((5,6,7,8-tetrahydrobenzo[4,5]thieno[2,3-d]pyrimidin-4-yl)oxy)acetohydrazide (5c)

**^1^HNMR chart (5c)**


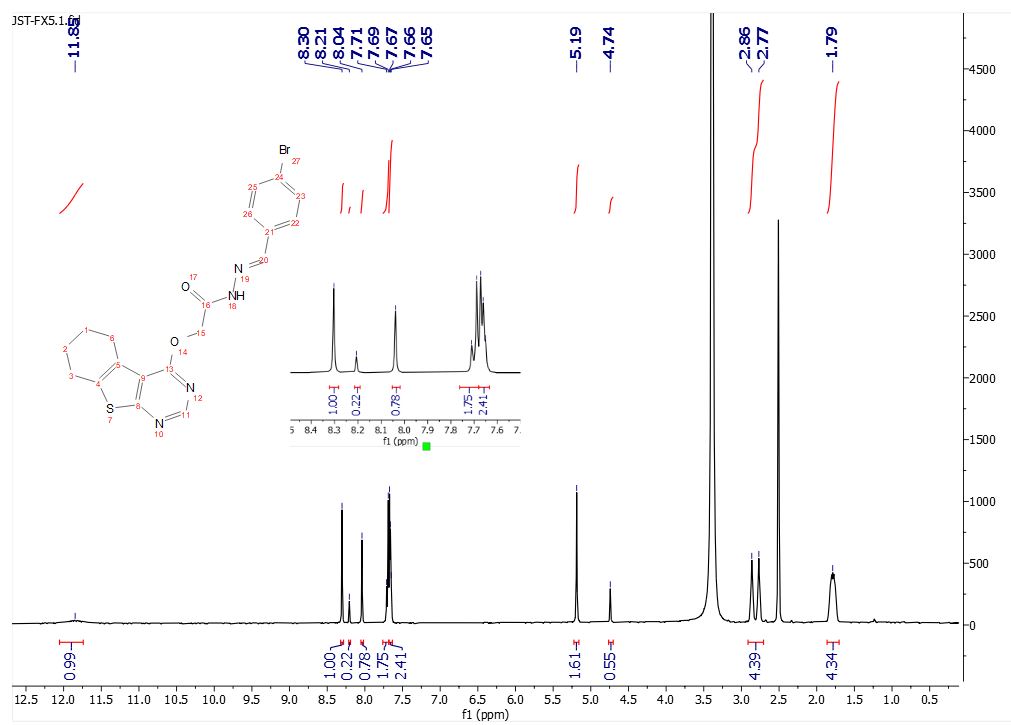


**IR chart (5c)**


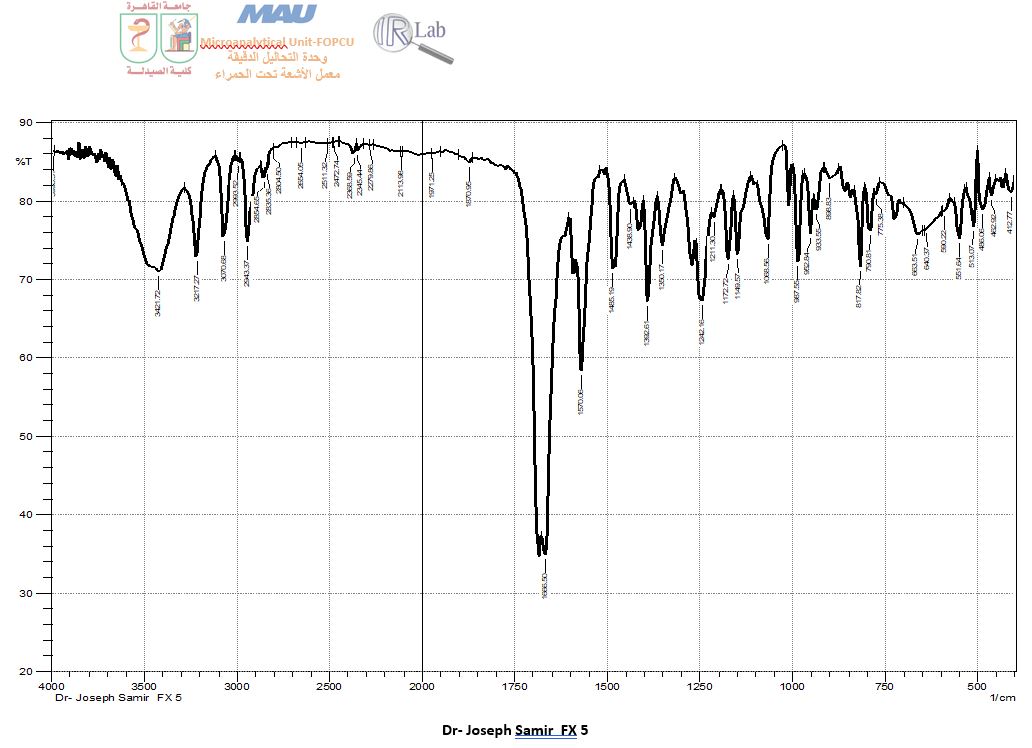


**Mass chart (5c)**

#### N'-(4-(Dimethylamino) benzylidene)-2-((5,6,7,8-tetrahydrobenzo [4,5] thieno[2,3-d] pyrimidin-4-yl) oxy) acetohydrazide (5d)

**^1^HNMR chart (5d)**


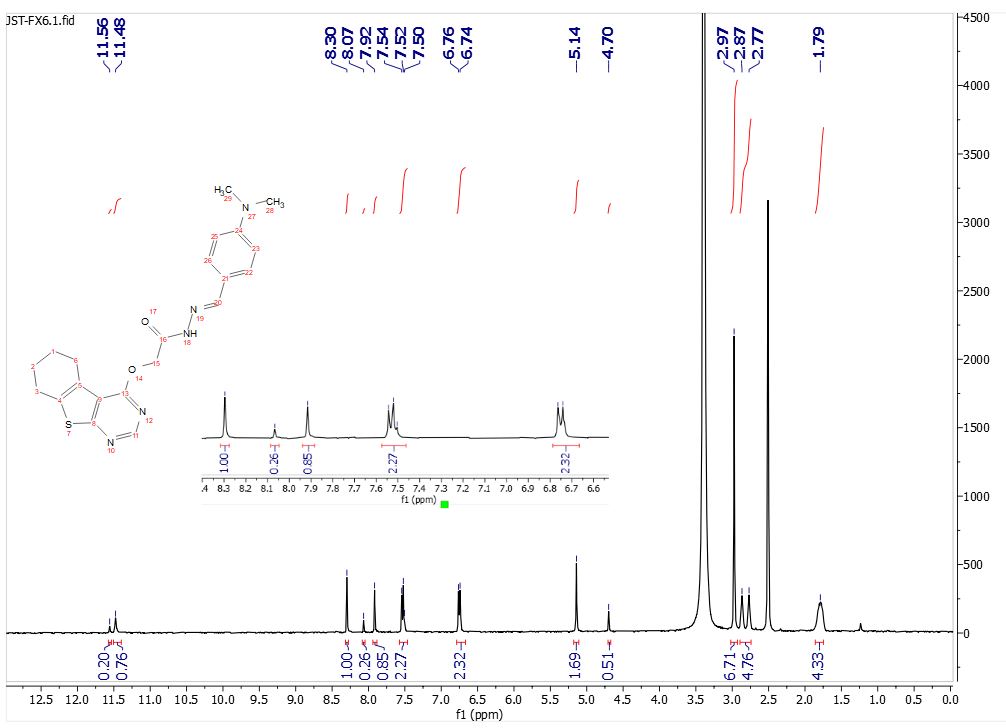


#### N-Phenyl-2-(2-((5,6,7,8-tetrahydrobenzo [4,5] thieno[2,3-d] pyrimidin-4-yl) oxy)acetyl hydrazine-1-carbothioamide (6a)

**^1^HNMR chart (6a)**


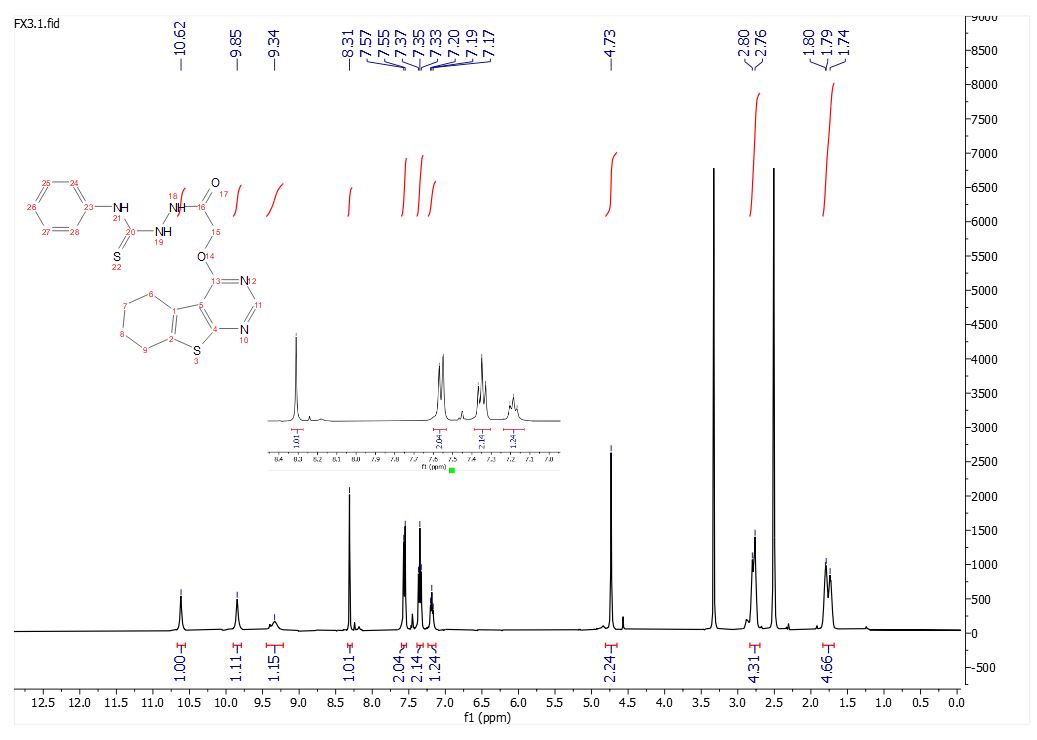


**IR chart (6a)**


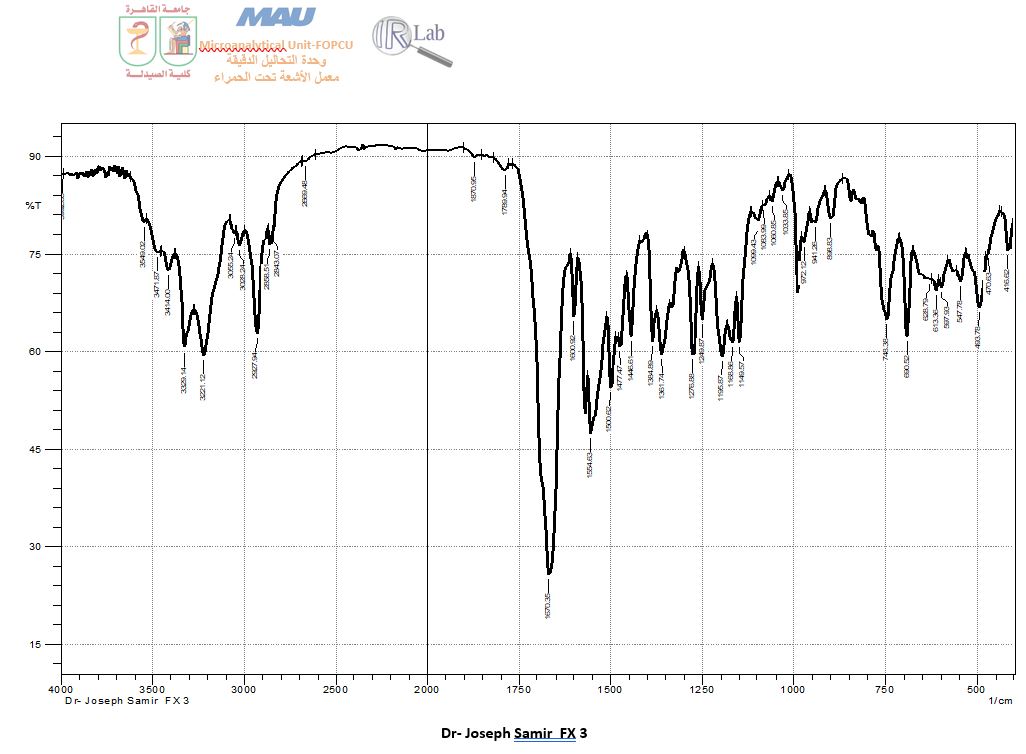


**Mass chart (6a)**


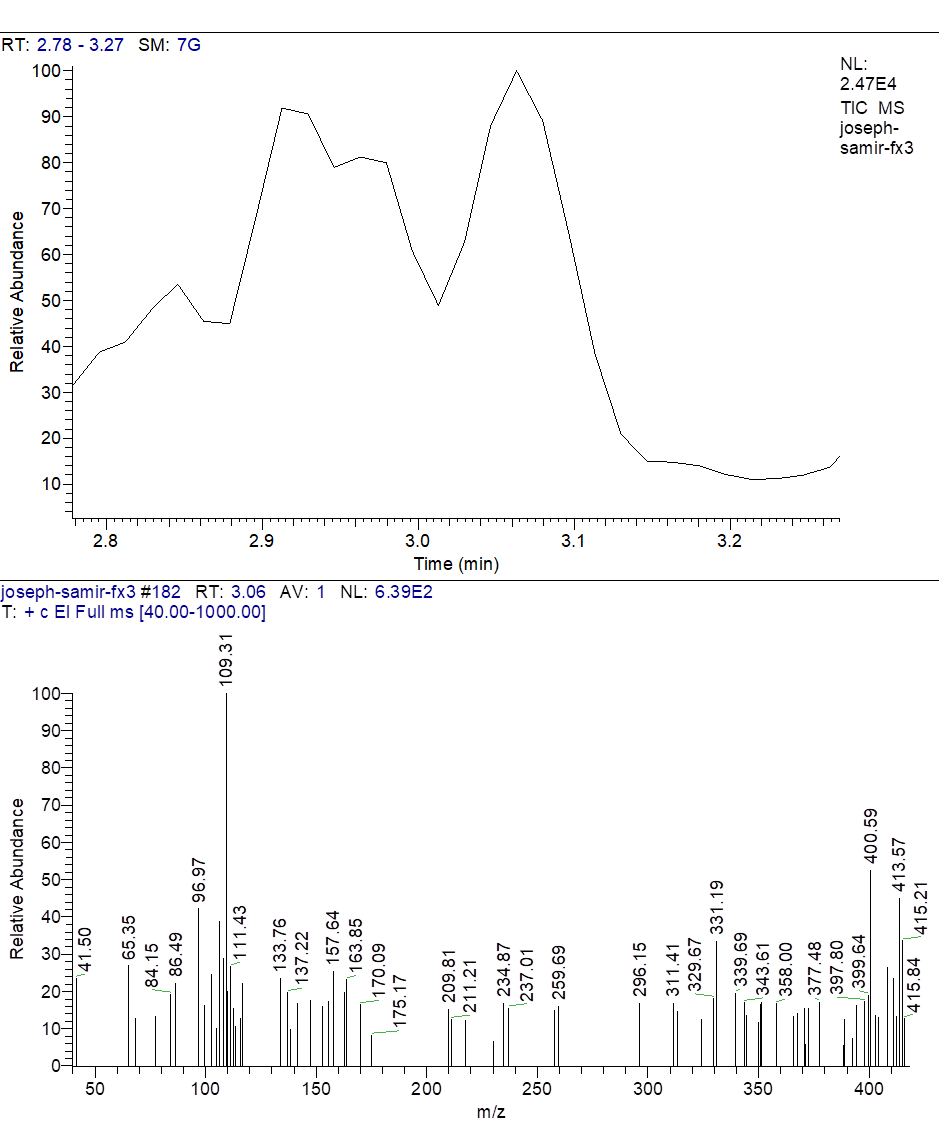


**^13^CNMR Chart (6a)**

**
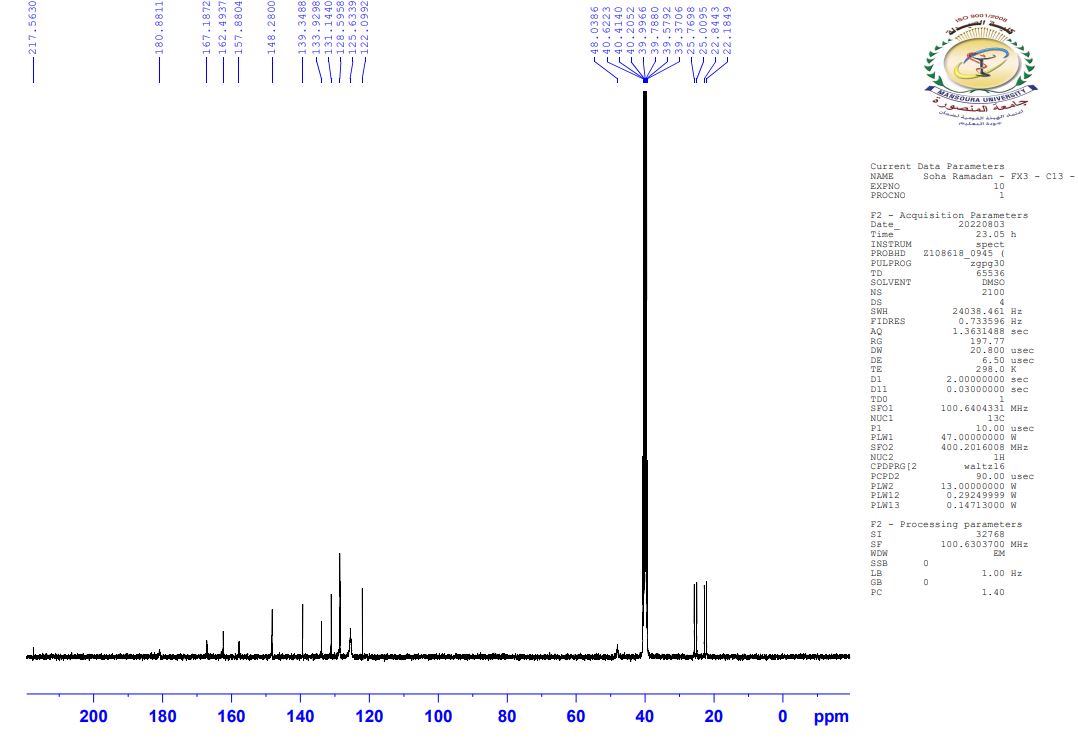
**

#### 4-Nitro-N'-(2-((5,6,7,8-tetrahydrobenzo [4,5] thieno[2,3-d] pyrimidin-4-yl) oxy) acetyl benzo hydrazide (6b)

####

**^1^HNMR chart (6b)**

####
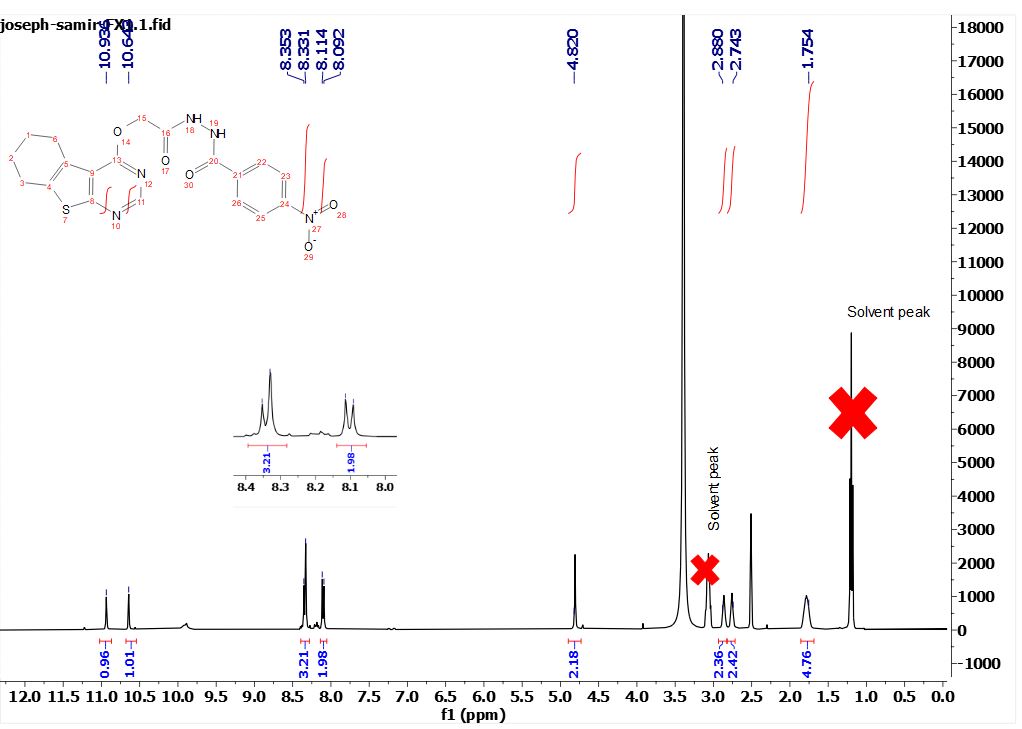


**D_2_O Proton NMR (6b)**


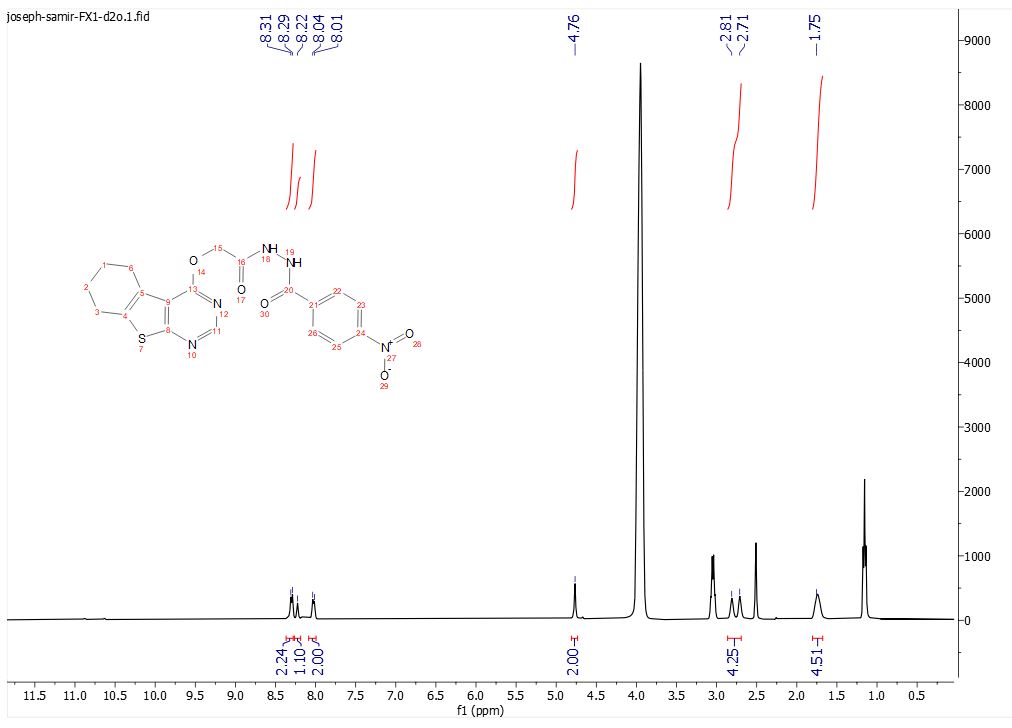


**IR chart (6b)**


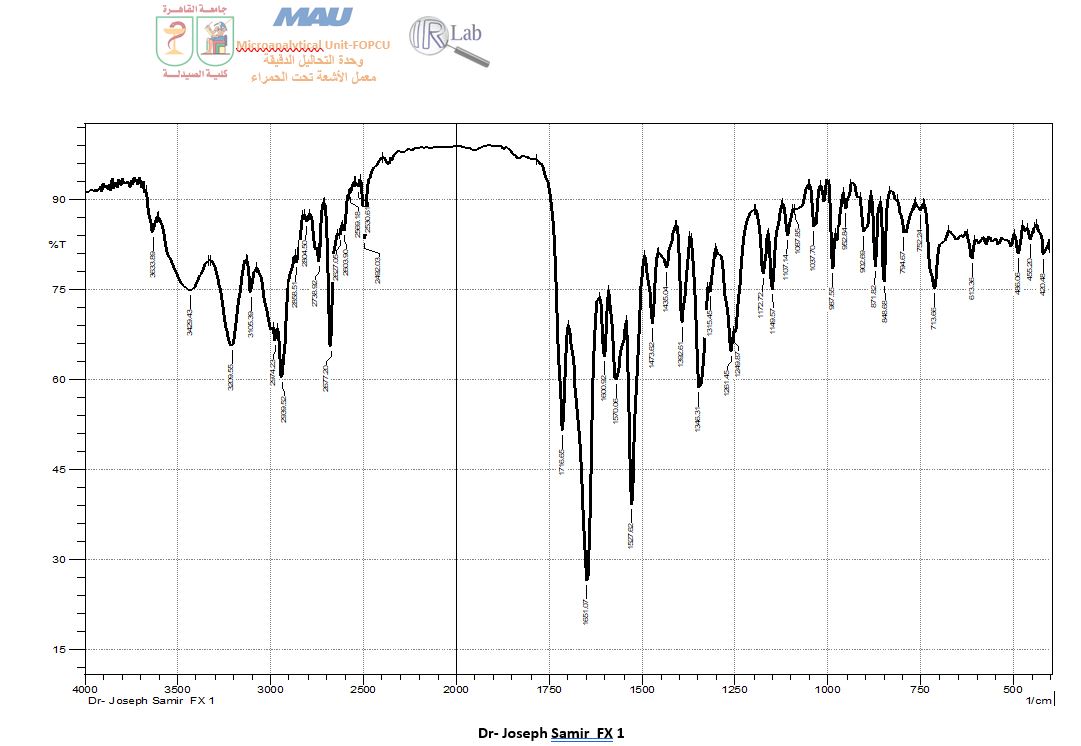


**Mass chart (6b)**

**
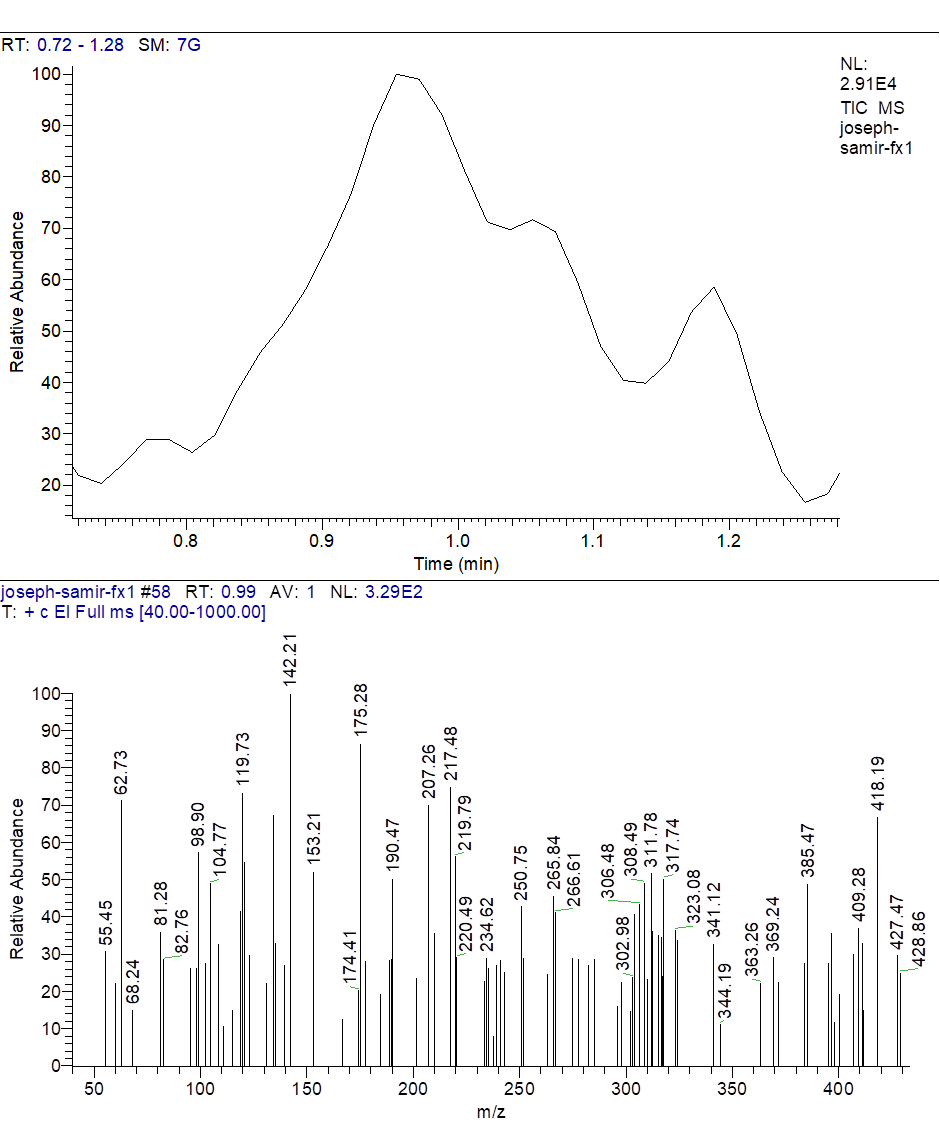
**

**^13^CNMR (6b)**

**
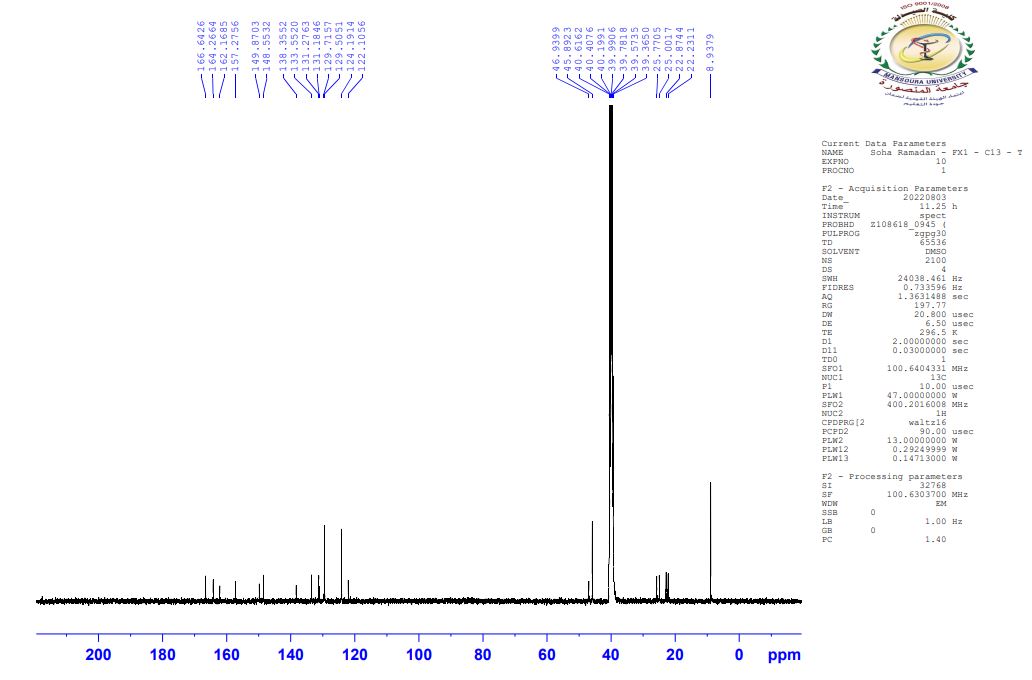
**

#### Phenyl-3-(5,6,7,8-tetrahydrobenzo [4,5] thieno[2,3-d) pyrimidin-4-yl) thiourea (9a)

**^1^HNMR chart (9a)**


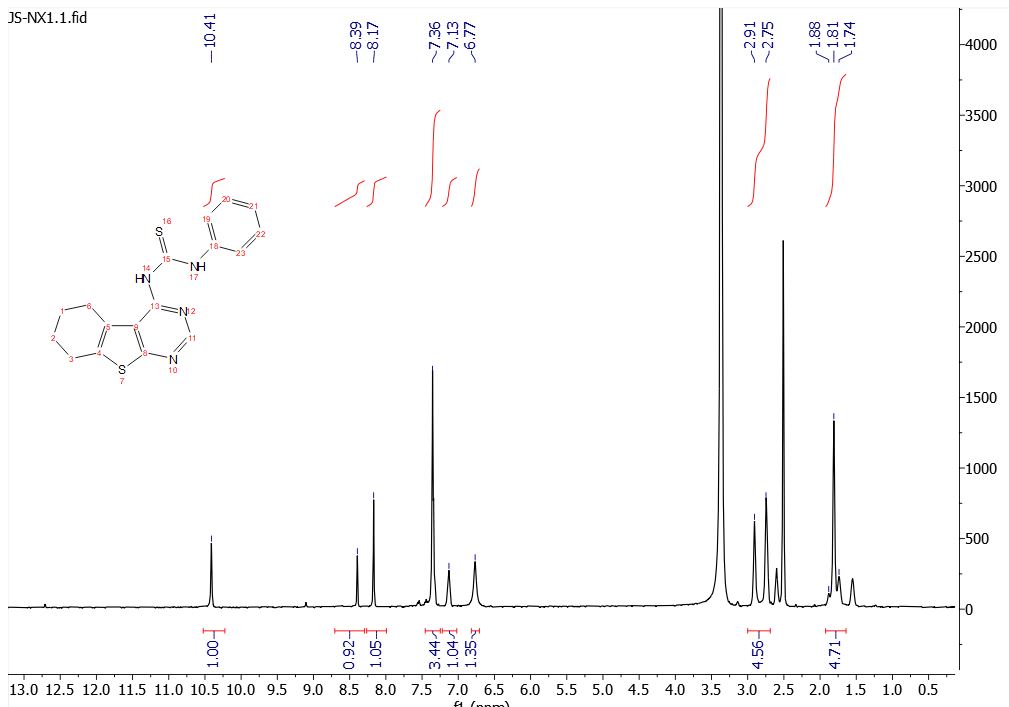


**IR chart (9a)**


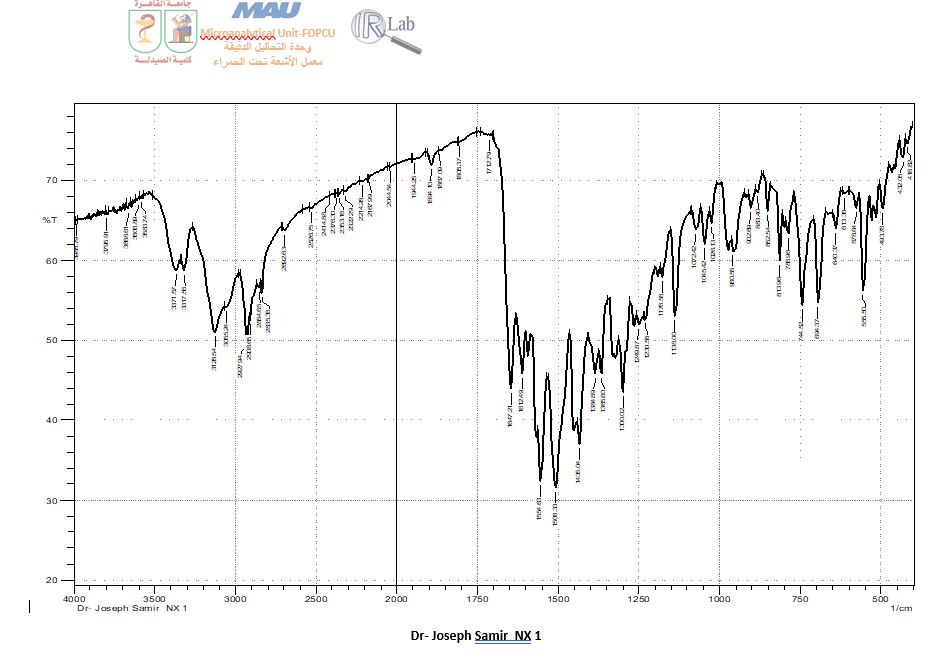


**Mass chart (9a)**


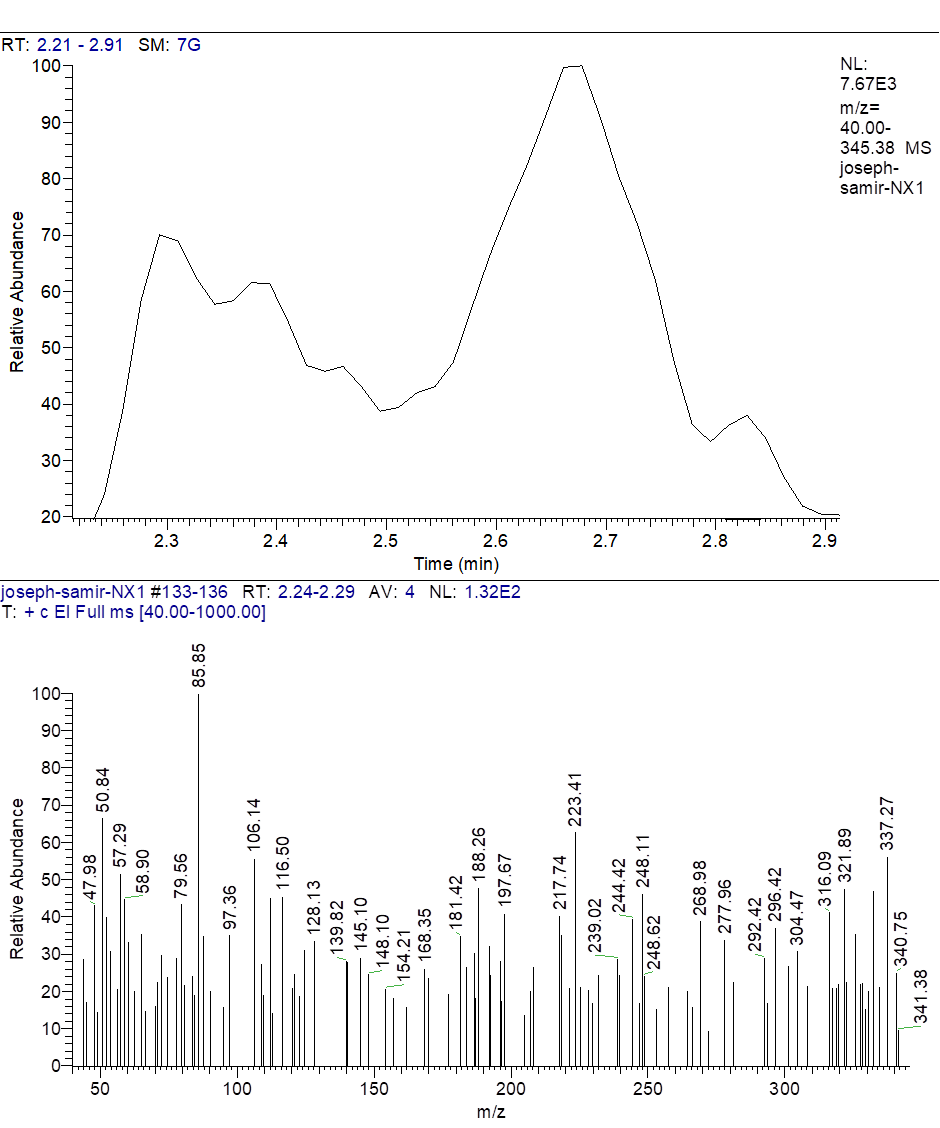


**^13^CNMR (9a)**

**
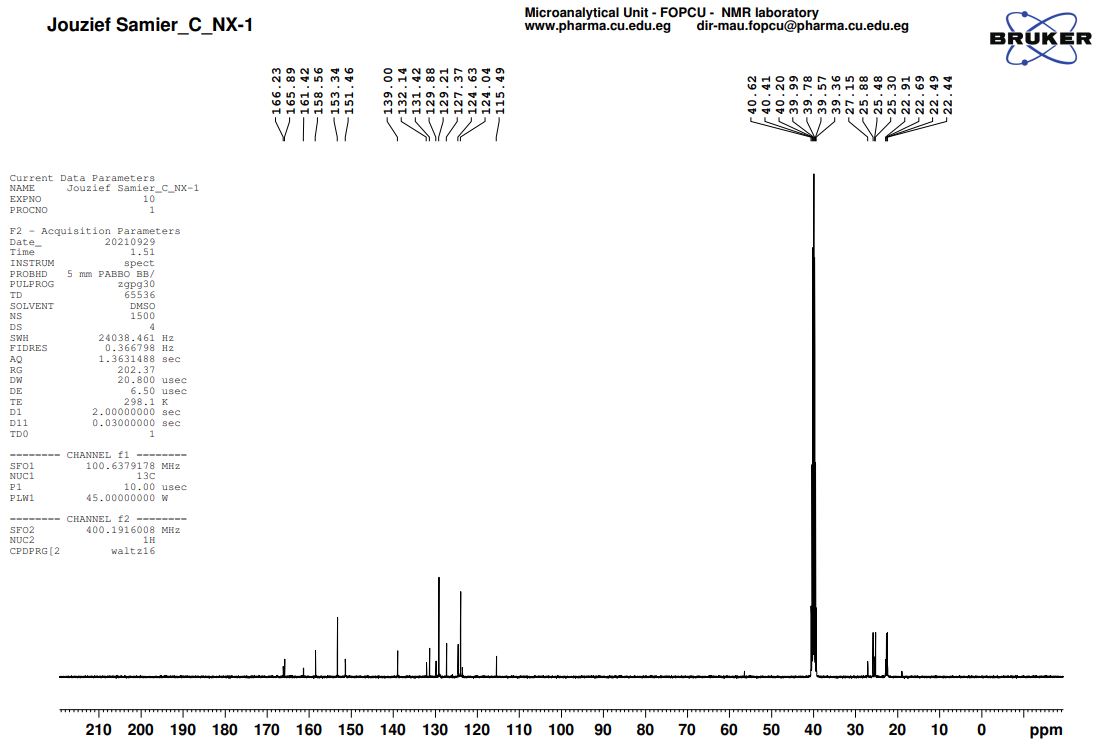
**

#### 1-(5,6,7,8-Tetrahydrobenzo[4,5]thieno[2,3-d]pyrimidin-4-yl)-3-(m-tolyl)urea (9b)

**^1^HNMR chart (9b)**


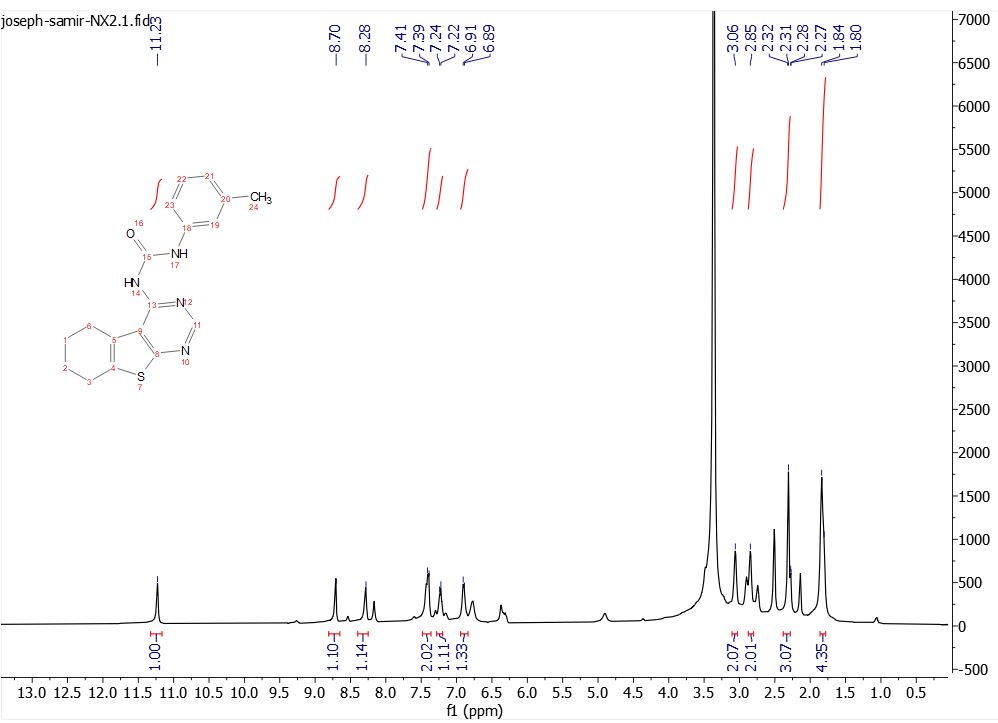


**D2O proton NMR(9b)**


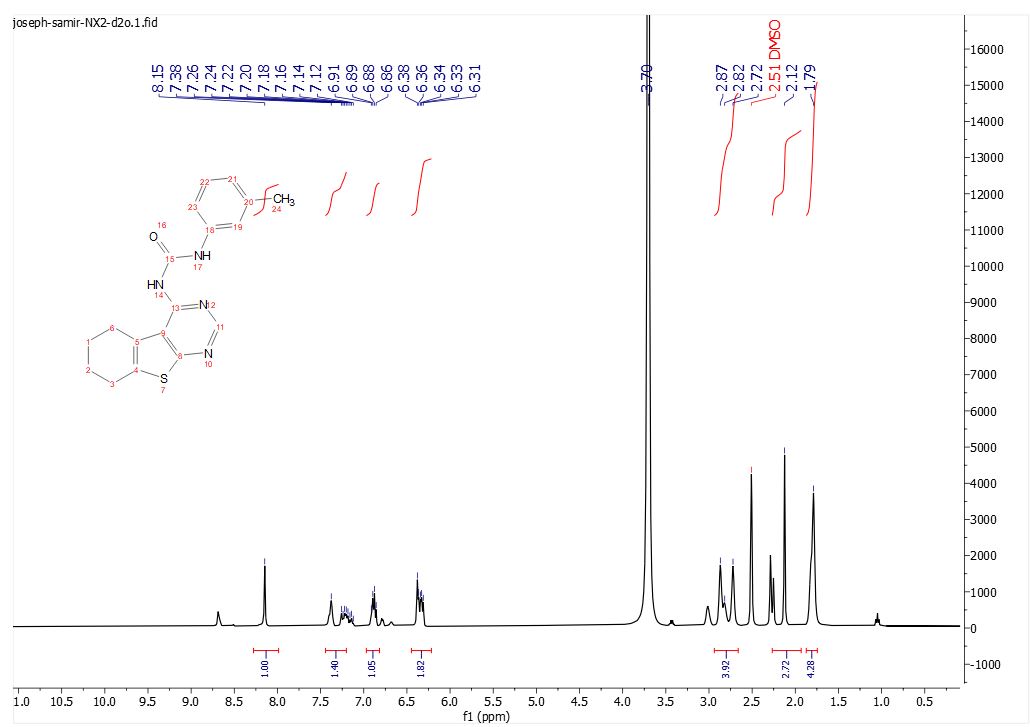


**IR chart (9b)**


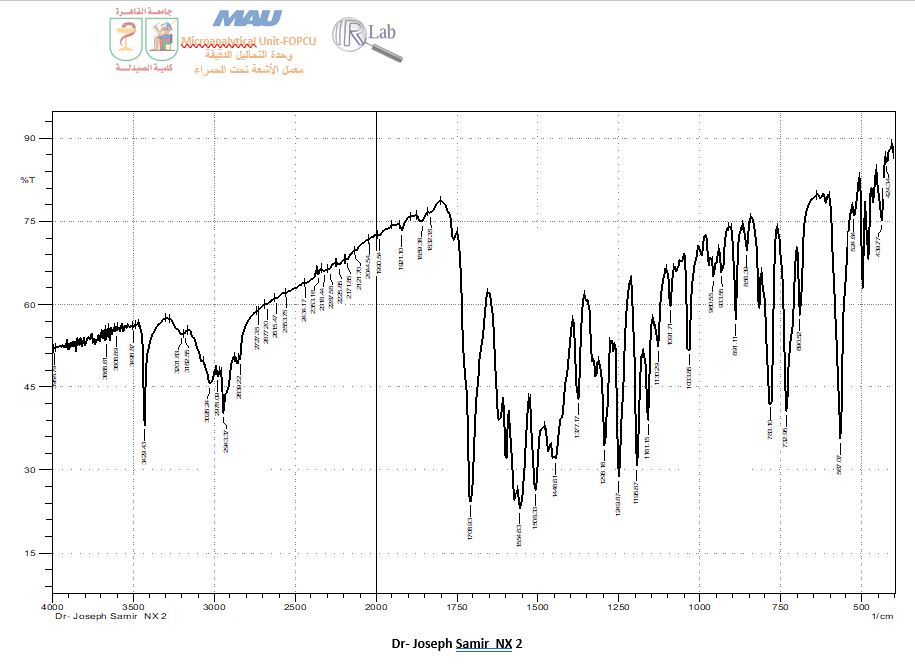


**Mass chart (9b)**


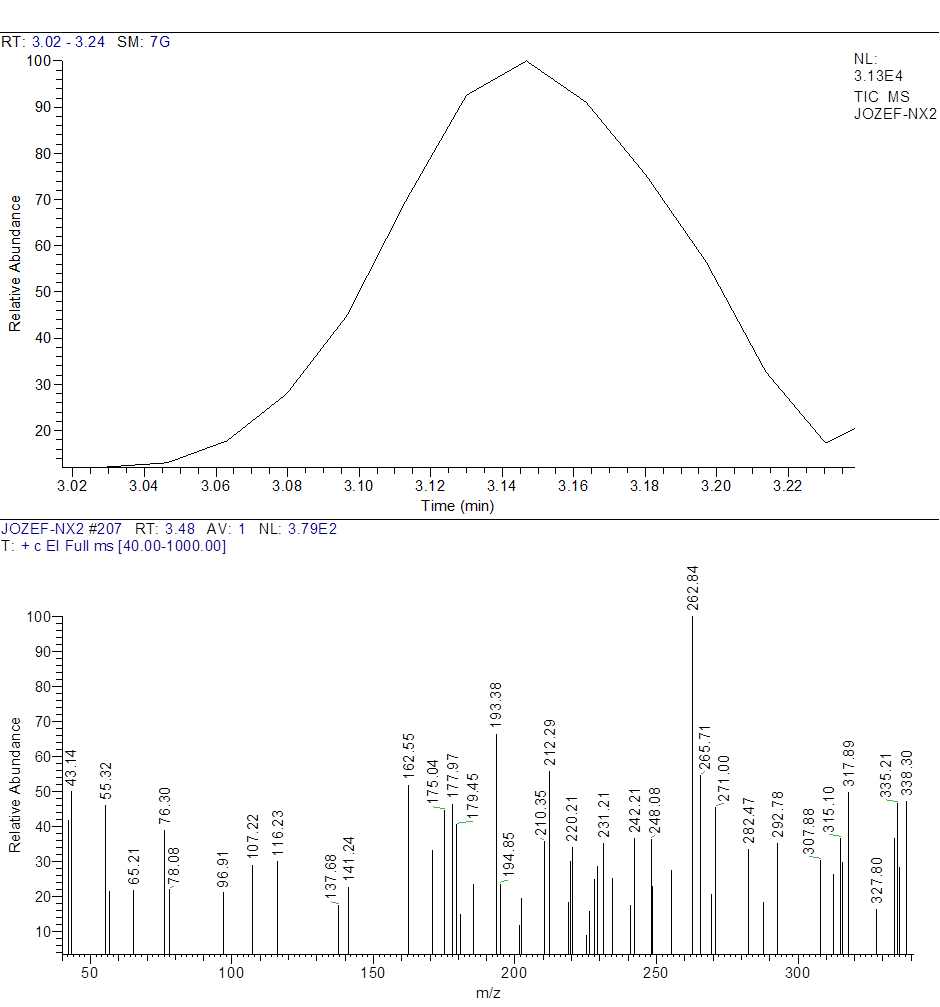


**^13^CNMR chart (9b)**


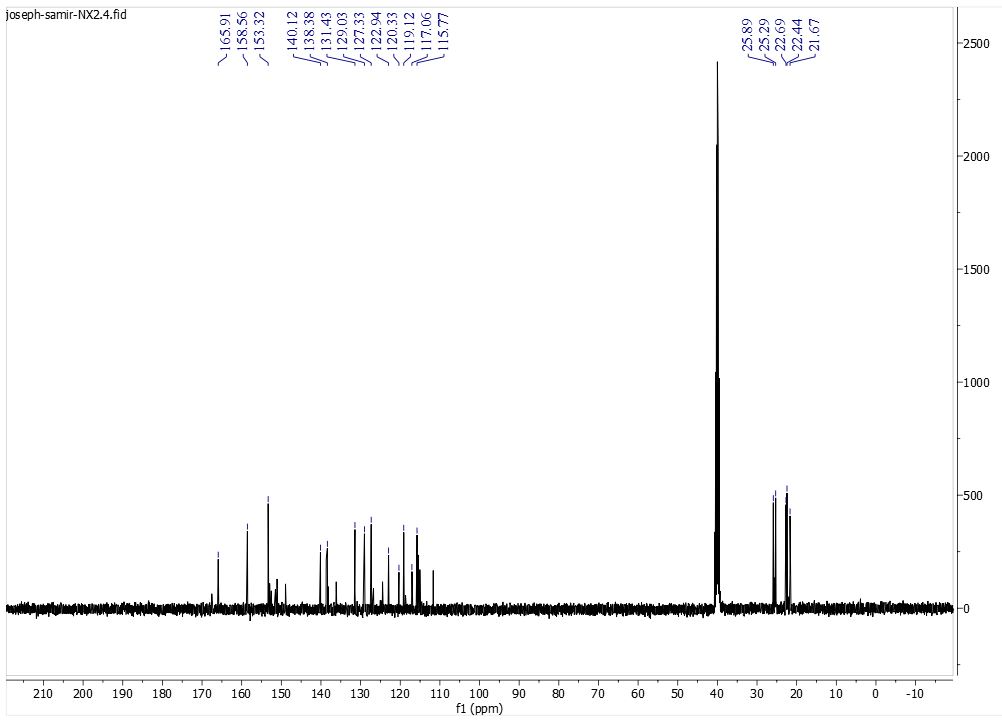


#### 1-(4-Chloro-3-(trifluoromethyl)phenyl)-3-(5,6,7,8tetrahydrobenzo[4,5]thieno[2,3-d]pyrimidin-4-yl)urea (9c)

**^1^HNMR chart (9c)**


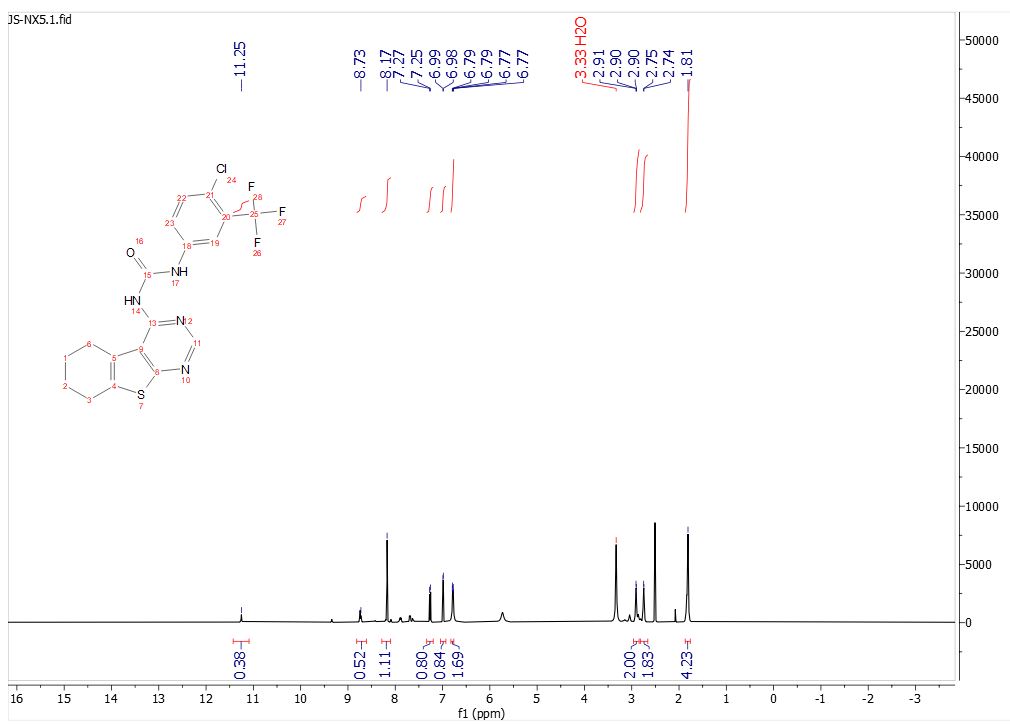


**D2O proton NMR(9c)**


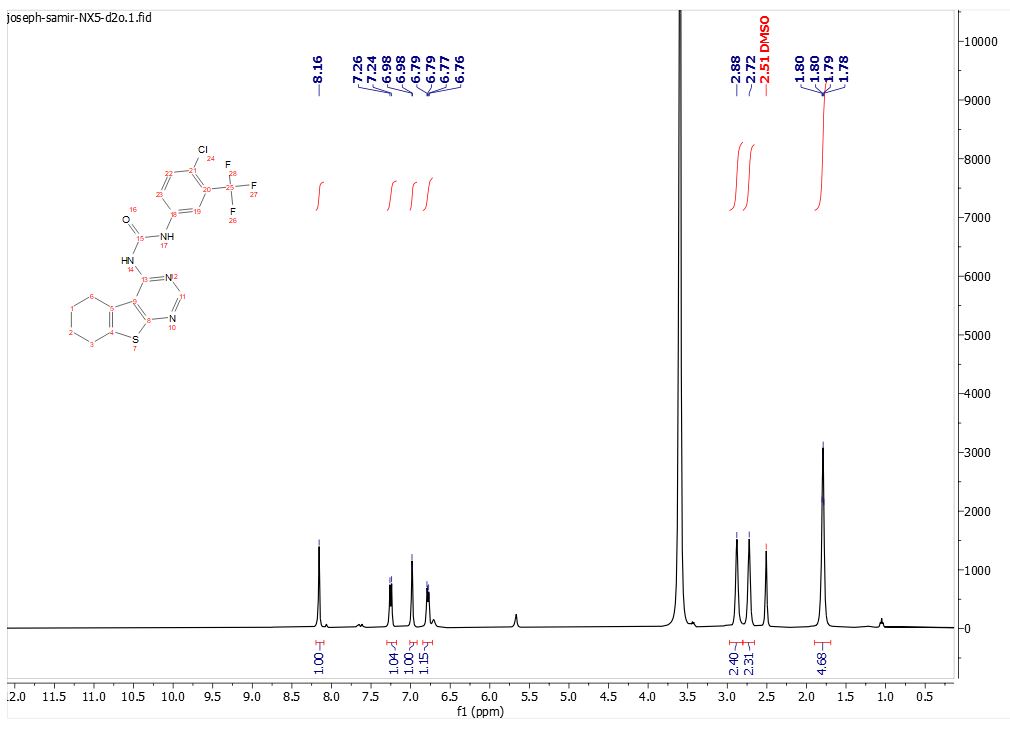


**IR chart (9c)**


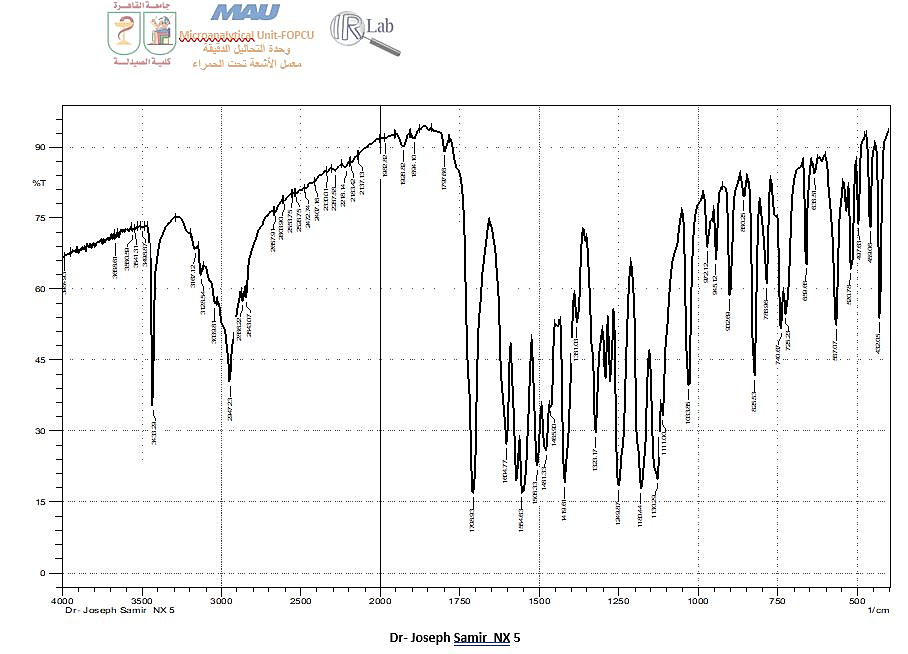


**Mass chart (9c)**

**Section 2. of supplementary materials (biological assessment of our target compounds).**

First part of biology is **Antiproliferative efficacy in vitro against NCI 60-cell line.**

The National Cancer Institute "NCI", NIH, Bethesda, Maryland, USA (www.dtp.nci.nih.gov) chose six of the final compounds for the Developmental Therapeutic Program (DTP), codes **(5a, 5b, 5c, 5d, 6a, 6b)**. The NCI screening service will favor compounds with drug-like modes of action based on computer-aided design. Selection The criterion for screening is the possibility of the submitted compounds to provide diversity to the NCI small molecule chemical collection. This screen employs 60 different Leukemia, melanoma, and additional tumor cell lines of the human from brain, kidney, lung, ovary, colon, breast, and prostate cancers. All compounds were picked based on their NCI codes. NSC: D-820696/1, NSC: D-820697/1, NSC: D-820698/1, NSC: D-820699/1, NSC: D-820700/1, NSC: D-820701/1. The initial 10 µM one dose percent inhibition assay was performed on the entire NCI 60 cell panel to examine the different chemotypes of this work. The results are presented as a percentage of cell growth on each of the 60 NCI cell line panels for each of the investigated substances. some examples of the results mention below.

#### N'-(4-(Dimethylamino) benzylidene)-2-((5,6,7,8-tetrahydrobenzo [4,5] thieno[2,3-d] pyrimidin-4-yl) oxy) acetohydrazide (5d)


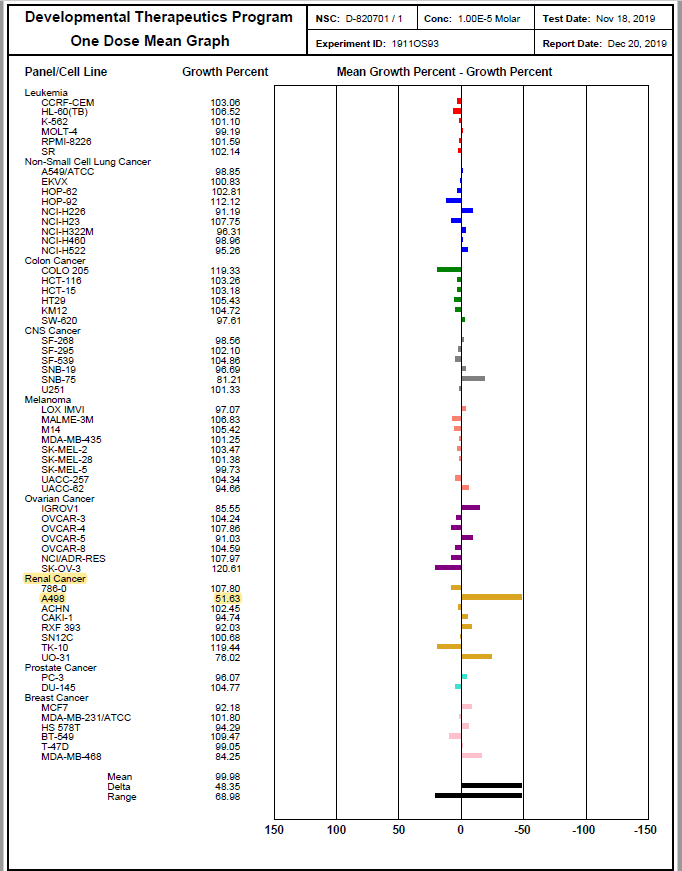

#### N-Phenyl-2-(2-((5,6,7,8-tetrahydrobenzo [4,5] thieno[2,3-d] pyrimidin-4-yl) oxy)acetyl hydrazine-1-carbothioamide (6a)


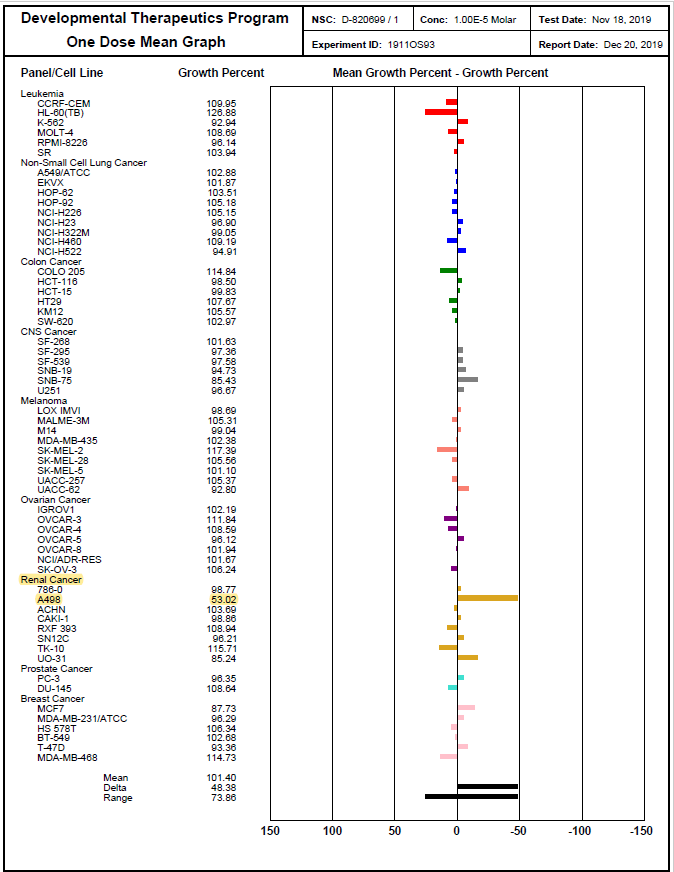

**Section 2.2** **biological evaluation Preliminary screening at 100 µM) a single dose Concentration)**

The GSK-3β tyrosine kinase tests were carried out at Thermo Fischer Scientific USA (www.thermofischer.com/select screen). The investigation was carried out to evaluate the GSK-3β inhibitory activity of the synthesized compounds. During the main reaction of the kinase, a single residue of serine, threonine, or tyrosine in a produced FRET-peptide receives gamma-phosphate of ATP through the biological test Z'-LYTE. In the secondary process, Non-phosphorylated FRET-peptides are identified and degraded by a site-specific protease. The phosphorylation of FRET-peptides inhibits the development reagent from cleaving them. FRET between the donor (coumarin) and acceptor (fluorescein) fluorophores on the FRET-peptide is disrupted by cleavage, while FRET is maintained by uncleaved, phosphorylated FRET-peptides. The percentage of enzymatic activity that the tested substances inhibited GSK-3β kinase was compared to a 100 µM as reference concentration of the kinase inhibitor (Staurosporine with IC_50_= 10 nM).

**Result enzyme assay for (6a=Js3) (9b=Nx2)**


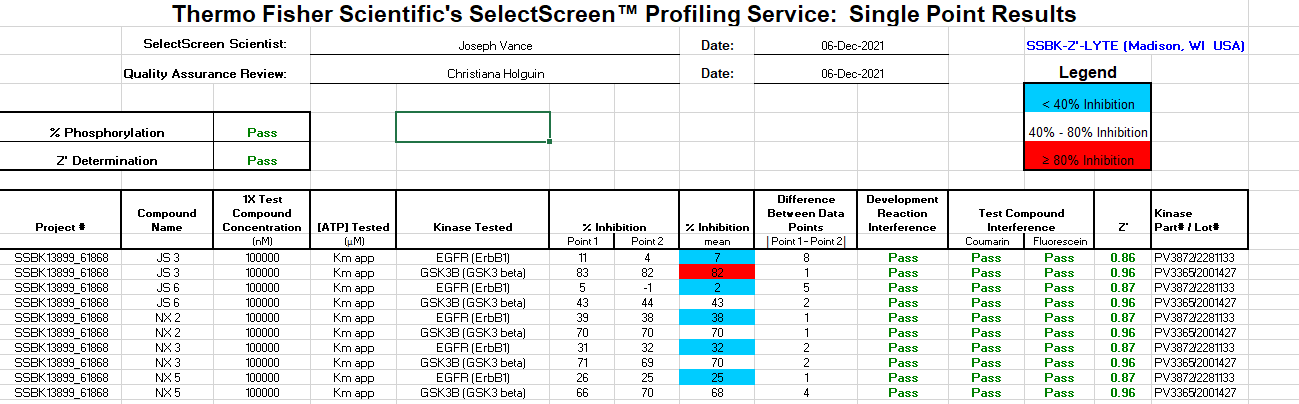


**Result enzyme assay for (6b=Fx1)**


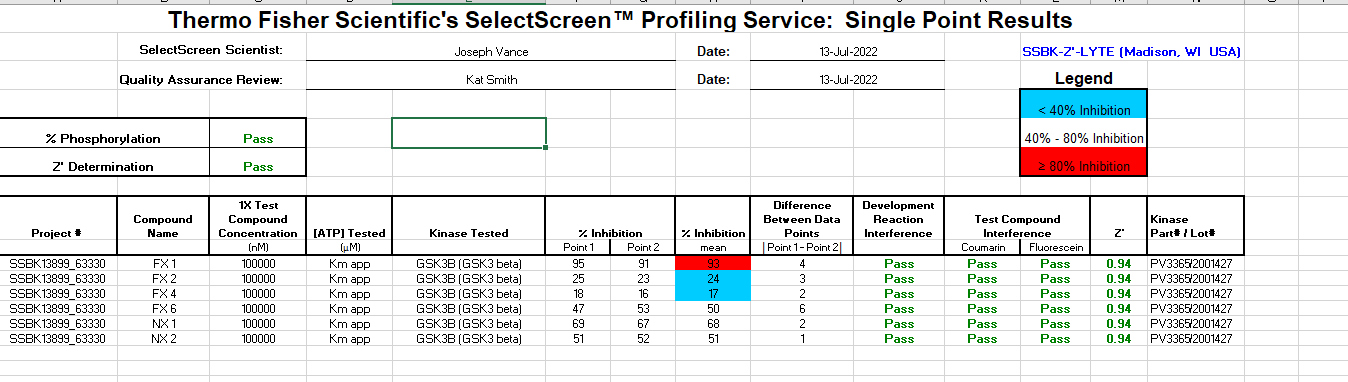


**Section 2.3 Evaluation of five dose inhibitory activity (IC50 .**

**Result for (6a=Js3) (9b=Nx2)**

**
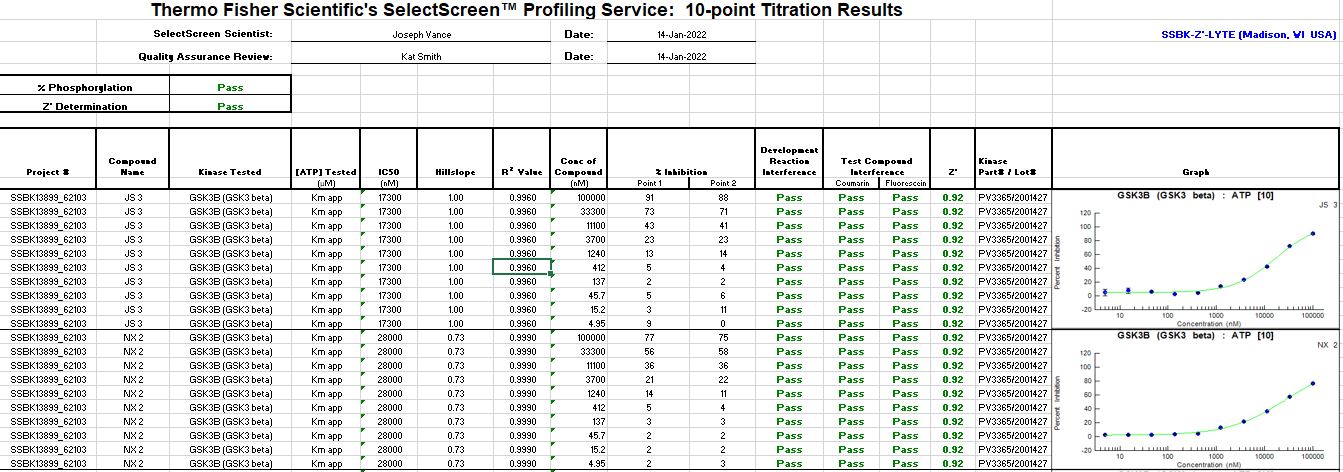
**

**Result for (6b=Fx1)**


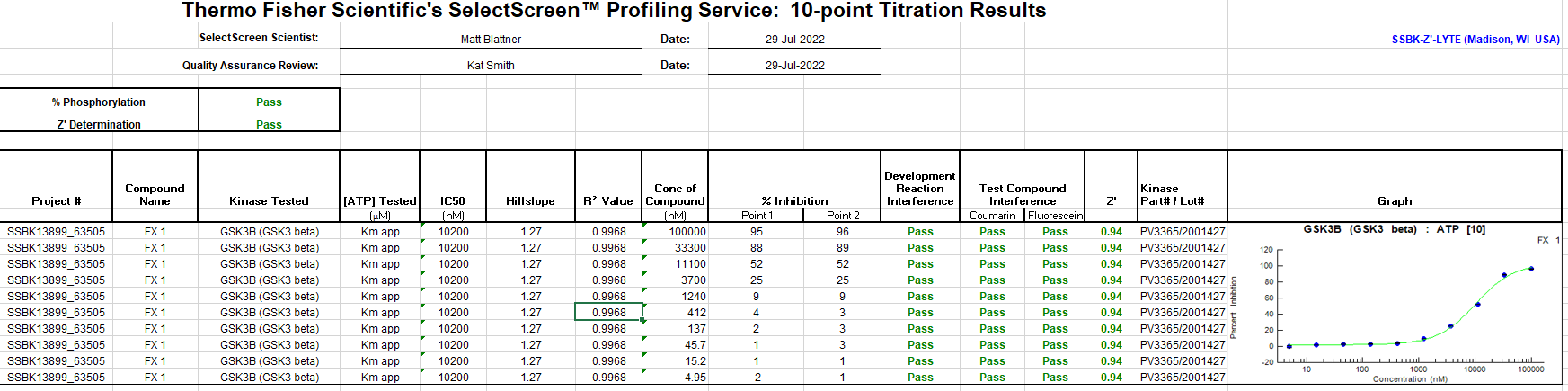

Supplement: Supplementary file 1 — Additional file 1. supplementary material. [file 13065_2023_1026_MOESM1_ESM.docx]
